# Modulation of IGF2 Expression in the Murine Thymus and Thymic Epithelial Cells Following Coxsackievirus-B4 Infection

**DOI:** 10.3390/microorganisms9020402

**Published:** 2021-02-15

**Authors:** Hélène Michaux, Aymen Halouani, Charlotte Trussart, Chantal Renard, Hela Jaïdane, Henri Martens, Vincent Geenen, Didier Hober

**Affiliations:** 1 GIGA-I3 Center of Immunoendocrinology, GIGA Research Institute, University of Liège, 4000 Liège, Belgium; hmichaux88@gmail.com (H.M.); charlotte.trussart@uliege.be (C.T.); ach.charlet@outlook.be (C.R.); hmartens@uliege.be (H.M.); 2 Laboratoire des Maladies Transmissibles et Substances Biologiquement Actives LR99ES27, Université de Monastir, 5000 Monastir, Tunisia; halouani.aymen@yahoo.com (A.H.); jaidanehela@yahoo.fr (H.J.); 3 Laboratoire de Virologie ULR3610, Université de Lille CHU Lille, F-59000 Lille, France

**Keywords:** thymus, thymic epithelial cell, type 1 diabetes, coxsackievirus B4, insulin-like growth factor 2, transcription, picornavirus

## Abstract

Coxsackievirus B4 (CV-B4) can infect human and murine thymic epithelial cells (TECs). In a murine TEC cell line, CV-B4 can downregulate the transcription of the insulin-like growth factor 2 (*Igf2*) gene coding for the self-peptide of the insulin family. In this study, we show that CV-B4 infections of a murine TEC cell line decreased *Igf2* P3 promoter activity by targeting a region near the transcription start site; however, the stability of *Igf2* transcripts remained unchanged, indicating a regulation of *Igf2* transcription. Furthermore, CV-B4 infections decreased STAT3 phosphorylation in vitro. We also showed that mice infected with CV-B4 had an altered expression of *Igf2* isoforms as detected in TECs, followed by a decrease in the pro-IGF2 precursor in the thymus. Our study sheds new light on the intrathymic regulation of *Igf2* transcription during CV-B4 infections and supports the hypothesis that a viral infection can disrupt central self-tolerance to insulin by decreasing *Igf2* transcription in the thymic epithelium.

## 1. Introduction

The presentation of self-peptides by thymic epithelial cells (TECs) is a process by which newly developed T-cells in the thymus are selected to be tolerant to self-antigens. This process plays an essential role in the central self-tolerance to neuroendocrine functions. Indeed, a defect in this process (e.g., loss of insulin expression) is the earliest event in the pathogenesis of autoimmune diseases such as type 1 diabetes (T1D) [1]. 

Insulin-like growth factor 2 (IGF2), which shares a high homology with insulin, is not only a self-peptide of the insulin family but also the dominant member of the insulin family in the thymus. Thus, IGF2 is more tolerated than insulin itself [1]. Besides, the IGF2 epitope competes with the major insulin epitope for major histocompatibility complex class II DQ8 (molecule conferring genetic susceptibility to T1D), and its presentation induces immunosuppressive cytokines in contrast to the insulin epitope [2]. Importantly, *Igf2*^−/−^ mice have decrease insulin tolerance [3]. Thus, IGF2 expression is required for the development of complete immune self-tolerance to insulin. Interestingly, IGF levels, including the IGF2 level in the blood, are low in T1D patients [4]. In line with these data, IGF2 has immunoregulatory functions as improving the immunosuppressive functions of regulatory T- or B-cells [5,6]. Additionally, *Igf2* is also defective in the thymus of a rat model of T1D [7]. To the best of our knowledge, no nonself-peptide of the insulin family has been discovered to date.

Viruses of the Enterovirus genus, especially coxsackievirus B (CV-B), are among the most important environmental factors linked to the onset of T1D [8]. These viruses are nonenveloped, and they have a positive-sense single-stranded RNA; they belong to the *Picornaviridae* family and are mainly transmitted via the orofecal route. The so-called diabetogenic strain CV-B4 E2 was isolated from a child with ketoacidosis after an acute T1D onset [9]. CV-B4, frequently detected in T1D patients, has a tropism for insulin-secreting β cells of the islets of Langerhans, and various mechanisms have been suggested to explain the induction of autoimmune T1D mediated by enteroviruses [8]. It has been investigated whether CV-B4 infects thymic cells and inhibits central self-tolerance toward insulin and pancreatic β cells. Following oral and intraperitoneal inoculation, CV-B4 can infect the thymus of Swiss albino and SJL/J mice, leading to abnormal T-cell precursor differentiation [10,11,12,13], a phenomenon also observed in murine Swiss albino and human thymic fetal organ cultures [14,15]. Besides, CV-B4 can induce a persistent infection of primary cultures of human TECs and modulate inflammatory cytokine expression [16]. Importantly, in the MTE4-14 cell line (a TEC cell line derived from neonatal mice [17]), CV-B4 drastically downregulates *Igf2* expression and persistent infection is established in this cell line. IGF1 (another member of the insulin family) is less affected by CV-B4 in this model [18].

The murine *Igf2* gene comprises three main promoters (*Igf2* P1, *Igf2* P2, and *Igf2* P3), resulting in three transcript isoforms differing only in their first exon in the 5′ untranslated region: *Igf2 Variant 3* (*Igf2* P3), *Igf2 Variant 1* (*Igf2* P2), and *Igf2 Variant 2* (*Igf2* P1) [19]. Although all *Igf2* transcripts are translated to the protein IGF2, differences exist, especially in the transcriptional and post-transcriptional regulation of the *Igf2* transcripts [20,21,22]. 

However, it has not been determined which are the *Igf2* transcripts and promoters modulated and which are the contributing factors affecting the decrease in *Igf2* mRNA expression in TECs following infection with CV-B4. Interestingly, signal transducer and activator of transcription 3 (STAT3) is a positive regulator of the *Igf2* promoter in humans and mice [23,24,25] and is not only essential for TECs development and survival [26,27,28] but also for the induction of antigen-specific T-cell tolerance [29].

Therefore, we investigated the pattern of expression of *Igf2* transcript isoforms and STAT3 phosphorylation in MTE4-14 cells infected with CV-B4. Furthermore, the effect of the virus on *Igf2* expression in TECs of outbred mice infected with CV-B4 in vivo has been investigated.

## 2. Materials and Methods

### 2.1. Cells and Virus

The murine thymic epithelial cell line MTE4-14, derived from C3H/J (*H-2^k^*) thymic neonatal lobes [17], was cultured in complete Dulbecco’s Modified Eagle Medium (DMEM) containing 4.5 g/L glucose (Gibco, Gaithersburg, MD, USA) supplemented with 10% heat-inactivated fetal calf serum (FCS; Gibco), 2 mM L-glutamine (Gibco), 0.1 µg/mL epidermal growth factor (EGF; Sigma-Aldrich, Saint Louis, MO, USA), 100 U/mL penicillin, and 100 µg/mL streptomycin (Gibco) [17,18]. Vero cells (provided by the Laboratory of Virology and Immunology, Giga, University of Liège) were cultured in DMEM containing 4.5 g/L glucose supplemented with 10% FCS, 100 U/mL penicillin, and 100 µg/mL streptomycin. CV-B4 E2, the diabetogenic strain of CV-B4, (provided by Ji-Won Yoon, Julia McFarlane Diabetes Research Center, Calgary, AB, Canada) was propagated using the HeLa cell line cultured in DMEM containing 4.5 g/L glucose supplemented with 10% FCS. Supernatants were collected 4 days after inoculation; following three freeze/thaw cycles, they were centrifuged at 2500× *g* for 10 min, filtered, and stored at −80 °C.

### 2.2. Mice Inoculation with CV-B4

Thirty-six female Swiss albino mice, aged 4–6 weeks, were purchased (Janvier Laboratories, Le Genest-Saint-Isle, France) and housed in groups of six. Mice were weighed daily and handled according to the general ethical guidelines with unlimited access to food and water. Mice were inoculated intraperitoneally with either 100 μL of CV-B4 E2 at 1.10^6^ TCID_50_/mL diluted in sterile Dulbecco’s Phosphate-Buffered Saline (DPBS, Thermo Fisher Scientific, Waltham, MA, USA) or 100 μL of the supernatant of mock-infected cells diluted in sterile DPBS, wherein these mice served as the control group. Six of each CV-B4-inoculated and mock-inoculated mice were sacrificed 2, 3, and 7 days postinfection (P.I.) for harvesting the thymus and pancreas. This study was approved by the Ethics Committee of University Hospital of Liège (Protocol n°13-1611).

### 2.3. TEC Isolation, Enrichment, and Immunostaining

The protocol has been described elsewhere [30,31]. Briefly, thymic lobes were cut into small pieces; residual blood and connective tissues were removed. The lobes were then digested for 15 min at 37 °C in Roswell Park Memorial Institute (RPMI) (Lonza, Basel, Switzerland) containing 500 µg/mL Liberase TL (Sigma-Aldrich) and 111 µg/mL DNase I from bovine pancreas (Sigma-Aldrich). Thymic fragments were mixed at the beginning and end of enzymatic digestion. The resulting supernatant was incubated for 5 min on ice in DPBS, supplemented with 1% FCS and 5 mM EDTA, pH of 7.3; the reaction was stopped by the addition of complete RPMI. The thymic cell suspension was then centrifuged and filtered; 50 × 10^6^ million cells were used for TECs enrichment with mouse CD45 microbeads (Miltenyi Biotec, Bergisch Gladbach, Germany), according to the manufacturer’s instructions. For immunostaining, CD45-negative enriched cells (TECs) and the total thymic cell suspension were incubated with 1:50 anti-CD16/CD32 Fc block (clone 93, eBioscience, Santa Clara, CA, USA) for 15 min at 4 °C in MACS buffer followed by staining with 1:100 anti-CD45 (clone 30F-11, BD Biosciences, Franklin Lakes, NJ, USA) and 1:100 anti-EpCAM (clone G8.8, eBioscience) simultaneously for 30 min. Samples were analyzed on a FACSCanto flow cytometer (BD Biosciences) using the FlowJo software (Tree Star, Ashland, OR, USA). The mean purity of the sorted CD45-negative cells was 80%.

### 2.4. Infection of MTE4-14 Cells with CV-B4

MTE4-14 cells seeded at 150,000 cells/well in 6-well flat-bottom plates were cultured in DMEM containing 4.5 g/L glucose supplemented with 10% heat-inactivated FCS, 2 mM L-glutamine, and 0.1 µg/mL EGF and incubated overnight at 37 °C. The culture medium was then discarded, and cells were inoculated with either 500 µL per well of DMEM (mock) or CV-B4 with a multiplicity of infection (MOI) of 0.05. This MOI was optimal to achieve the downregulation of the *total Igf2* expression, in a window period of 3 days, with minimal need for observing the cytopathic effects (Supplementary Figure S1). Alternatively, 20,000 MTE4-14 cells were seeded in 96-well flat-bottom plates and inoculated with 100 µL of CV-B4 at MOI ranging from 0.05 to 5. Cell counting was performed in one well before inoculation, ensuring similar MOI between experiments. After a 90-min incubation, cells were rinsed with DMEM alone and incubated with complete DMEM without antibiotics. Mock-infected cells were treated similarly in all experiments. At 1, 2, and 3 days P.I, adherent viable cells were scraped and rinsed using PBS; subsequently, they were centrifuged and stored for further molecular analysis.

### 2.5. Modified TCID_50_ Titration Assay

The tissue culture infectious dose 50 (TCID_50_) of CV-B4 in Vero cells was determined using the modified Reed and Muench limiting dilution assay as previously described [32]. The TCID_50_ assay is used to quantify viral titers by determining the concentration at which 50% of the infected cells display a viability of 50%. Briefly, after an incubation period of 7 days with various dilutions of CV-B4, cells were incubated for 3 h with 3-(4,5-Dimethyl-2-thiazolyl)-2,5-diphenyl-2H-tetrazolium bromide reagent (MTT; Sigma-Aldrich). The formazan crystals were then dissolved in DMSO, and absorbance was measured at 550 nm. TCID_50_ values (limiting dilution corresponding to 50% of viability) were obtained using the V_50_ parameter of the Boltzmann sigmoidal function.

### 2.6. Flow Cytometry

For MTE4-14 flow cytometric analysis, cells were harvested with 5 mM EDTA in DPBS for 15 min at 37 °C and rinsed with 10% FCS before the addition of the Fc block as described above. Staining was performed using 1:40 antimouse CD126 APC (clone D7715A7, BioLegend, San Diego, CA, USA) or APC rat IgG2b, κ isotype control (BioLegend).

### 2.7. One-Step and Two-Step CV-B4 RNA Detection Using PCR

One-step RT-PCR was performed using SuperScript III One-Step RT-PCR System with Platinum Taq DNA Polymerase (Thermo Fisher Scientific). Reactions were performed following the manufacturer’s instructions and contained 500 ng of RNA and 100 nM of 007 and 008 primers [13], which were subjected to the following conditions: 50 °C for 30 min for cDNA synthesis, followed by one cycle at 94 °C for 2 min; 38 cycles at 94 °C for 30 s, 60 °C for 30 s, and 68 °C for 30 s and ended at 72 °C for 10 min. PCR products were visualized using gel electrophoresis. A seminested PCR of 35 cycles was performed using the negative PCR products with GoTaq G2 DNA polymerase as described above. For the two-step CV-B4 RNA detection, reverse transcription was performed as described above using a reaction volume of 10 µL comprising 1 µM reverse primer 007 (5′-ATTGTCACCATAAGCAGCCA-3′) for the positive strand of CV-B4 or 1 µM forward primer 008 (5′-GAGTATCAATAAGCTGCTTG-3′) for negative strand detection of CV-B4. PCR amplification was then performed with 100 nM of 007 and 008 primers. PCR parameters were as follows: 95 °C for 2 min, followed by 25 cycles at 95 °C for 30 s, 60 °C for 30 s, and 72 °C for 30 s. PCR was ended after 5 min at 72 °C. PCR products were analyzed on an agarose gel, and the PCR product size was 412 bp. A seminested PCR of 35 cycles was performed as described above using GoTaq G2 DNA polymerase with the negative PCR products and internal primers 006 (5′-TCCTCCGGCCCCTGAATGCG-3′) and 007. The seminested PCR product size was 155 bp.

### 2.8. Reverse Transcription, Endpoint, and Real-Time Quantitative PCR

Total RNA was extracted using the NucleoSpin RNA Kit (Macherey-Nagel, Düren, Germany), according to the manufacturer’s instructions. Alternatively, RNA was extracted with TRIzol Reagent (Thermo Fisher Scientific), according to the manufacturer’s instructions. The RNA concentration was measured using the Nanodrop ND-1000 (Thermo Fisher Scientific). An A_260/280_ ratio > 1.8 was considered acceptable. Reverse transcription was performed using 200–500 ng of total RNA with the Transcriptor First Strand cDNA Synthesis Kit (Roche, Basel, Switzerland), 60 µM of random hexamer, and 6.25 µM of oligo(dT)_18_ primer. A control without reverse transcriptase enzyme was included as control to verify the absence of genomic DNA contamination. Endpoint PCR was performed using GoTaq G2 Flexi DNA Polymerase (Promega, Madison, WI, USA) with 25 mM MgCl_2_, 1× Green Buffer, 0.625 U GoTaq G2 polymerase, 10–25 ng cDNA, 200 µM dNTPs, and 200 nM of each forward and reverse primer (Table 1). Cycling conditions were as follows: 95 °C for 2 min, followed by 35 cycles at 95 °C for 30 s, 60 °C for 30 s, and 72 °C for 30 s and ended at 72 °C for 5 min. PCR products were visualized on an agarose gel.

Real-time PCR was performed on an iCycler iQ (BioRad, Hercules, CA, USA) for 40 cycles with Takyon No Rox SYBR 2× MasterMix blue dTTP (Eurogentec, Seraing, Belgium) and 200 nM of each primer (Eurogentec) (Table 1). Each qPCR reaction was ended considering the melting curve with a ramp of 0.5 °C from 55 to 95 °C to control single PCR product amplification. A nontemplate control was used as the negative control in each reaction. Gene expression values were calculated according to the comparative Ct normalized to *Hprt* [18] and presented as a fold change with respect to mock (2^−ΔΔCt^) or as a relative value (2^−ΔCt^).

### 2.9. Igf2 P3 Nluc Plasmid Construction and Site-Directed Mutagenesis

The 342-bp *Igf2* P3 promoter sequence containing the murine *Igf2* P3 proximal promoter [33,34] was synthesized using GeneArt Gene Synthesis (Thermo Fisher Scientific). The transcription start site (TSS) sequence was localized using the Eukaryotic Promoter Database [35]. The *Igf2* P3 promoter sequence was then cloned into the Nanoluciferase expressing promoterless vector pNL1.2[NlucP] (Promega) using *NheI* (5′) and *EcoRV* (3′) restriction sites with GeneArt Gene Synthesis. The resulting plasmid was called *Igf2* P3 Nluc. Site-directed mutagenesis or promoter deletions were performed with the Q5 Site-Directed Mutagenesis Kit (New England Biolabs, Ipswich, MA, USA) with 500 nM of each forward and reverse primer (Table 1) and 50 ng of plasmid. PCR was performed at the following conditions: 98 °C for 30 s, followed by 25 cycles at 98 °C for 10 s, for 30 s at 65–71 °C (Table 1), and 72 °C for 150 s and ended at 72 °C for 2 min. The site-directed mutagenesis PCR product was visualized on 1% agarose gel and then treated with the KLD Enzyme Mix (New England Biolabs) prior to transformation into the *E. coli* strain DH5-α competent cells. Plasmids were extracted with NucleoSpin Plasmid Transfection-grade (Macherey-Nagel), and their concentrations were measured using Nanodrop ND-1000. All plasmids were sequenced using Sanger sequencing (Giga-Genomics).

### 2.10. Dual-Luciferase Reporter Assay

In 96-well plates, 20,000 MTE4-14 cells were transfected per well during seeding with a mix of 20 µL of 100 ng of pGL3 plasmid (Promega), 100 ng of *Igf2* P3 Nluc (or empty vector or constructs derived from *Igf2* P3 Nluc), and 0.5 µL of GENIUS DNA Transfection Reagent (Westburg, Leusden, The Netherlands) diluted in DMEM. After overnight incubation at 37 °C, cells were then inoculated with CV-B4 or DMEM only as described above. Nanoluciferase activity and firefly luciferase activity were analyzed 1 and 2 days P.I. using the Nano-Glo Dual-Luciferase Reporter Assay System (Promega), according to the manufacturer’s instructions. Bioluminescence was analyzed with FilterMax F5 (Molecular Devices, San Jose, CA). Normalized luciferase activity was calculated as the ratio of Nanoluciferase activity to firefly luciferase activity for each sample and was then normalized with the empty vector for mock and CV-B4-infected cells. In each experiment, the relative mock value was set to 100%.

### 2.11. mRNA Stability Assay

Actinomycin D (Sigma-Aldrich) was prepared at 1 mg/mL in ethanol and used at a final concentration of 5 µg/mL. The mRNA half-life of *Igf2 V3* was estimated with linear regression in each experiment using the relative *Igf2 V3* value (2^−ΔCt^) (normalized to the value of the vehicle control for each time point) with the formula $2^{-\Delta \mathrm{Ct}\;\left(Igf2V3-Hprt\right)}=f\left(time\;of\;actinomycin\;D\;treatment\right)$ for mock or CV-B4-infected cells.

### 2.12. SDS-PAGE and Western Blot

Cells were lysed using the RIPA buffer containing protease and phosphatase inhibitor mini tablets cocktail (Pierce, Thermo Fisher Scientific) and stored at –20 °C for further use. Total protein concentrations were determined using the BCA Protein Assay Kit (Pierce, Waltham, MA, USA); 10 µg (or 50 µg for IGF2 detection) of proteins were loaded on the 12% SDS-PAGE gel and then transferred to the PVDF membrane (GE Healthcare, Chicago, IL, USA). Membranes were blocked with 5% *w/v* bovine serum albumin (BSA) in Tris-buffered saline with Tween-20 (TBS-T) for 1 h at 20–22 °C, followed by overnight incubation at 4 °C with primary antibodies (Table 2) diluted in 5% BSA TBS-T. Membranes were then incubated with antirabbit or antimouse secondary antibody coupled to horseradish peroxidase (all from Cell Signaling Technology) diluted at 1:1000 in 5% BSA TBS-T for 1 h at 20–22 °C. Chemiluminescence was visualized with Pierce ECL Western blotting substrate (Pierce) and acquired on ImageQuant LAS4000 (GE Healthcare). The relative quantification of bands was analyzed with the ImageJ software. Each band was individually selected with the rectangular selection and “Gels” function, followed by quantification of peak areas of obtained histograms. Data were acquired as arbitrary area values. The ratios were obtained by normalization of arbitrary values of protein of interest to the corresponding bands of loading control (GAPDH or β Tubulin). Recombinant mouse IGF2 (500 ng, R&D Systems, Minneapolis, MN, USA) was used as a positive control for mature IGF2 detection.

### 2.13. Statistical Analyses

Statistical analyses were performed using GraphPad Prism 8.0 (San Diego, CA, USA). All data were analyzed for normality distribution using the Shapiro–Wilk test. The unpaired two-tailed *t*-test and ratio two-tailed paired *t*-test were used to compare between-group differences for in vivo and in vitro experiments, respectively. One-way ANOVA was used to compare differences between time points for in vivo and in vitro experiments. Alternatively, the Wilcoxon test was used when paired data failed to pass the normality test. *p* values ≤ 0.05 were considered significant.

## 3. Results

### 3.1. Decrease in Igf2 Transcripts and Pro-IGF2 Expression Following CV-B4 Infection of MTE4-14 Cells

**Figure 1:**
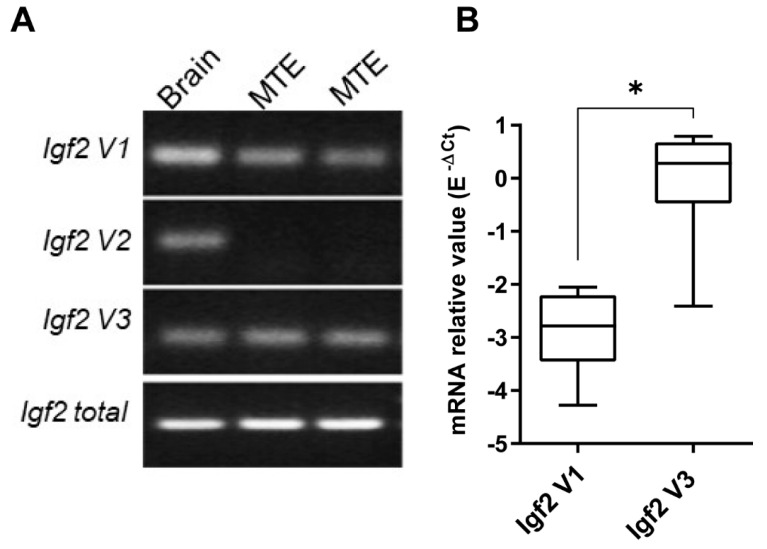
Insulin-like growth factor 2 (*Igf2*) mRNA isoforms expression in MTE4-14 cell line. (**A**) RT-PCR of *Igf2 V1* (90 bp), *Igf2 V2* (98 bp), *Igf2 V3* (68 bp), and *total Igf2* (107 bp). Mock samples from two independent experiments are represented; the murine brain is used as a positive control. The full-length gel is presented in Supplementary Figure S2B. (**B**) Relative mRNA expression of *Igf2 V1* and *Igf2 V3* in day-matched mock MTE4-14 cells; *n* = 6. Relative expression is normalized to *Hprt* with E^−ΔCt^ formula (E represents efficiency amplification for *Igf2 V3* and *Igf2 V1*); box-and-whisker plots extend from minimum to maximum values with lines at medians. Student’s *t*-test, * *p* < 0.05.

*Igf2* mRNA transcripts expressions were determined in mock-infected mice MTE4-14 (Figure 1A) using RT-PCR with specific primer pairs for each transcript (Supplementary Figure S2A). Among *total Igf2* transcripts, we detected *Igf2 V1* and *Igf2 V3* mRNAs in MTE4-14 cells after 3 days of culture (Figure 1A); *Igf2 V3* mRNA was dominant and, on average, 815-fold greater than *Igf2 V1*, suggesting a higher basal activity of the *Igf2* P3 promoter than the *Igf2* P2 promoter (Figure 1B). In addition, *Igf2 V1* mRNA was undetectable on day 1 or day 2 of culture (data not shown).

As an additional characterization of MTE4-14 cells, we investigated the autoimmune regulator (*Aire*) expression, but the results were inconclusive. Furthermore, the expression of *Ins2* (type 2 insulin, the insulin gene predominantly transcribed in the murine thymus) is not detected in this cell line [18].

**Figure 2:**
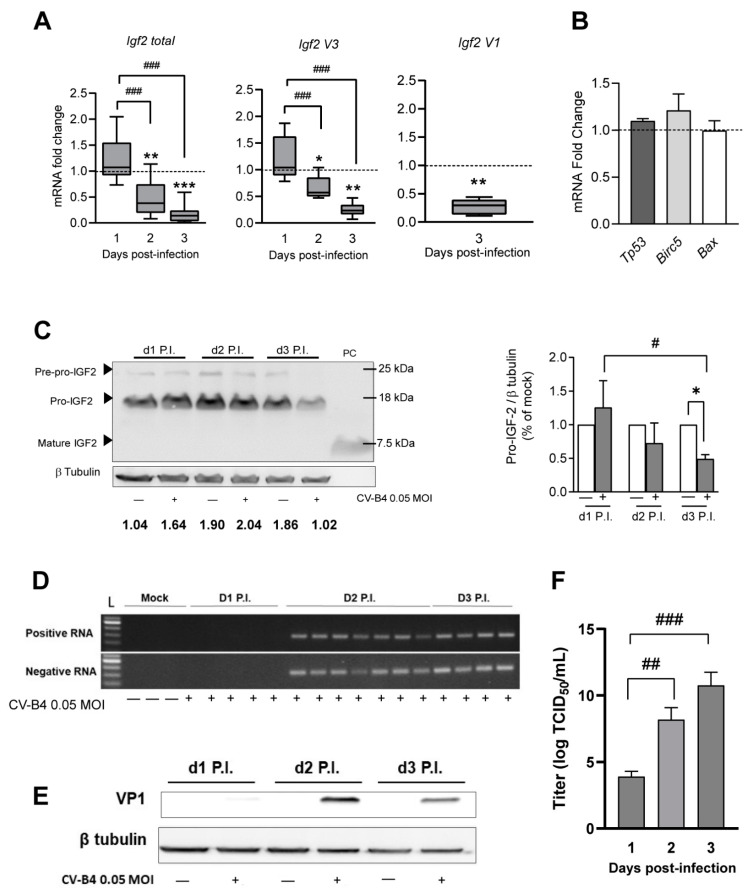
Effect of Coxsackievirus B4 (CV-B4 infection) on Igf2 in MTE4-14 cells. (**A**) Fold change of mRNA expression of Igf2 transcripts in CV-B4-infected cells relative to matched mock-infected cells; *n* = 6–12. Mock samples are represented as a dashed line set at y = 1; box-and-whisker plots (CV-B4-infected cells) extend from minimum to maximum values, with lines at medians. (**B**) Fold change of mRNA expression of *Tp53*, *Birc5*, and *Bax* in CV-B4-infected cells relative to matched mock-infected cells on day 3 P.I. Mock samples are represented as a dashed line set at y = 1; data are represented as the mean of fold change ± SEM; *n* = 3. (**C**) Left panel, Western blot analysis of IGF2 and its precursors in CV-B4-infected cells (+) and mock-infected cells (−). PC, purified mature IGF2. Representative full-length blots of IGF2 and β-tubulin are presented in Supplementary Figure S3D. Right panel, relative quantification of pro-IGF2 in CV-B4-infected cells (gray histograms) normalized to matched mock-infected cells (white histograms); *n* = 3. The histogram represents the mean of fold change ± SEM. (**D**) Agarose gel electrophoresis of amplicons specific to the positive and negative strands of the CV-B4 genome (155 bp), amplified by seminested strand-specific RT-PCR, using MTE4-14 cells infected with CV-B4 (MOI = 0.05); mock samples served as negative controls. Full-length gels are presented in Supplementary Figure S3E. (**E**) Western blot analysis of VP1 in CV-B4_-_infected MTE4-14 cells (+) or mock-infected cells (−). Representative full-length blots of VP1 and β-tubulin are presented in Supplementary Figure S3F. Data are representative of three independent experiments. (**F**) Viral titer of CV-B4 (MOI = 0.05) in MTE4-14-infected cells; *n* = 3–5. The mean of TCID_50_/mL ± SEM is shown. (**A**–**F**) CV-B4-infected cells with MOI = 0.05. (**A**–**C**) Ratio paired *t*-test, *** *p* < 0.001, ** *p* < 0.01 and * *p* < 0.05; (**A**,**C**,**F**) one-way ANOVA, # *p* < 0.05, ## *p* < 0.01, and ### *p* < 0.001.

During the CV-B4 infection (MOI = 0.05), RT-qPCR results showed a gradual decrease in *total Igf2* levels on day 2 and 3 P.I. (Figure 2A). Following testing at other MOIs, we observed a trend for a gradual decrease in *total Igf2* mRNA associated with an increase in MOI (Supplementary Figure S3A). Among *Igf2* mRNA transcripts, both median mRNA expression of *Igf2 V3* and *Igf2 V1* (representing a minor part of *Igf2* transcripts) decreased on day 3 P.I. in CV-B4-infected cells. Median *Igf2 V3* mRNA expression was 77% lower and 72% lower for *Igf2 V1* mRNA (Figure 2A).

The mRNA levels of the cell cycle regulator *Tp53* and the apoptosis regulators *Birc5* and *Bax*, previously shown to be altered by enteroviruses or by other positive-sense single-stranded RNA viruses [36,37,38], were unchanged during CV-B4 infection (Figure 2B). Moreover, we noticed an intriguing increase in *Igf2 V3* (as for the relative level of *total Igf2*) in mock-infected cells between 1 and 3 days P.I. (Supplementary Figure S3B). The IGF2 protein level and its precursors in MTE4-14 cells were investigated using Western blot. As shown above in in vivo experiments, mainly pro-IGF2 was detected with a similar molecular weight, and no mature IGF2 was observed in mock- and CV-B4-infected cells. In CV-B4-infected MTE4-14 cells, pro-IGF2 decreased gradually (*p* = 0.0036), especially on day 3 P.I., where an average decrease in 51% was observed in CV-B4-infected cells (*p* = 0.0116) (Figure 2C). A concomitant increase in CV-B4 replication and production was observed with a decrease in Igf2 expression (Figure 2D–F). Nevertheless, compared with mock-infected cells, cell viability was not impaired in CV-B4-infected cells until day 3 P.I. (Supplementary Figure S3C).

### 3.2. CV-B4 Infection Decreases Igf2 P3 Promoter Activity

The issue of the reduced activity of *Igf2* promoters in the decrease in *Igf2* transcripts in MTE4-14 cells infected with CV-B4 has been addressed. The transcriptional regulation of *Igf2 V3* was assessed as a main concern, by investigation of *Igf2* P3 promoter activity with CV-B4 infections. Indeed, *Igf2 V1* (encoded by the *Igf2* P2 promoter) represents only a weak part of *Igf2* transcripts in MTE4-14 cells and was detected on day 3 of culture, in contrast to *Igf2* V3, which was detected on days 1–3 of culture. Therefore, in the rest of this study, the impact of CV-B4 infection on *Igf2* transcripts dependent on the *Igf2* P2 promoter was not investigated.

**Figure 3:**
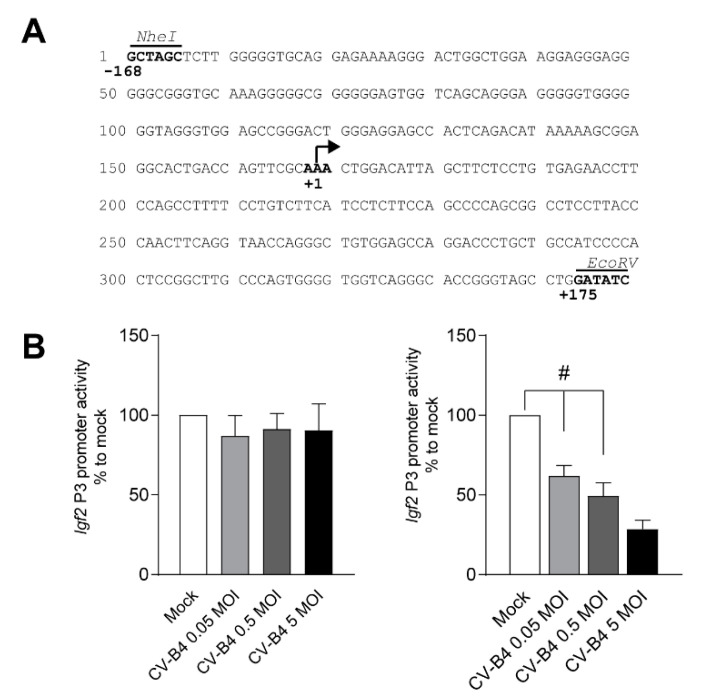
Effect of CV-B4 infection on *Igf2* P3 promoter activity in MTE4-14. (**A**) Sequence of the murine *Igf2* P3 promoter (−168/+175) https://epd.epfl.ch (accessed on 9 December 2020). Restriction sites *NheI* and *EcoRV* are indicated above in italics. The transcription start site (TSS) is represented by an arrow at +1. (**B**) Nanoluciferase relative activity of the *Igf2* P3 promoter (−168/+175) after 1 (left panel) and 2 days postinfection (P.I.) (right panel). The analysis is described in the Methods section. The mean of the relative dual-luciferase activity normalized to mock is represented as ± SEM. (**B**) One-way ANOVA, # *p* < 0.05.

To investigate *Igf2* P3 promoter activity, we cloned the *Igf2* P3 promoter (−168/+175 relative to the TSS) upstream of a Nanoluciferase reporter vector (Figure 3A). Although *Igf2* P3 promoter activity was unchanged on day 1 P.I., *Igf2* P3 promoter activity was 38% lower on day 2 P.I. in CV-B4-infected cells (MOI = 0.05, *p* = 0.0004). The loss of *Igf2* P3 promoter activity elevated with increasing CV-B4 MOIs (Figure 3B).

Given the fact that mRNA levels depend on mRNA transcription level and mRNA degradation, we decided to examine whether CV-B4 can impair the post-transcriptional regulation of *Igf2* transcripts. To this end, we used actinomycin D on day 2 P.I. and analyzed *Igf2 V3* mRNA expression using RT-qPCR. By inhibiting mRNA transcription, actinomycin D allows the measurement of mRNA half-life reflecting mRNA degradation. No difference in *Igf2 V3* mRNA half-life between mock- and CV-B4-infected cells was observed, even after 10 h of treatment. Of note, the average *Igf2 V3* mRNA half-life was estimated at 9.43 h in mock-infected cells (Supplementary Figure S4A). The *total Igf2* mRNA level was also analyzed after 10 h with actinomycin D treatment on day 2 P.I. A similar mRNA half-life was obtained in mock-infected cells, whereas no decrease in mRNA half-life was observed in CV-B4-infected cells (Supplementary Figure S4B). Owing to the lack of *Igf2 V1* expression on day 2 P.I., its mRNA stability was not assessed.

Together, these results indicate that CV-B4 does not play a significant role in the post-transcriptional level of *Igf2* expression and show that a decrease in *Igf2 V3* mRNA is associated with a decrease in the *Igf2* P3 promoter activity.

### 3.3. Impact of CV-B4 Infection on Specific Regions of the Igf2 P3 Promoter

**Figure 4:**
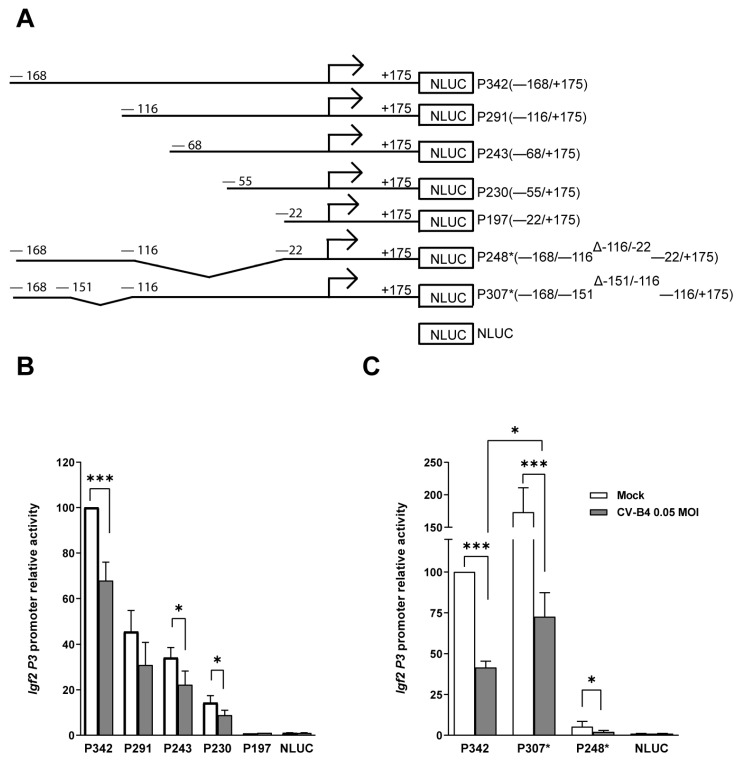
5′ Unidirectional Deletion analysis of *Igf2* P3 promoter in MTE4-14 cells Infected with CV-B4. (**A**) Schematic representation of the *Igf2* P3 promoter constructs in the Nanoluciferase vector; the arrow represents the TSS. (**B**,**C**) Nanoluciferase relative activity of *Igf2* P3 promoter constructs on day 2 P.I. in CV-B4 (MOI = 0.05) or mock-infected cells transfected with the indicated *Igf2* P3 promoter construct. The mean of the relative dual-luciferase activity normalized to mock is represented as ± SEM. Mock-infected cells transfected with the full *Igf2* P3 promoter (—168/+175) are set at 100% in each experiment; *n* = 4–16. Ratio paired *t*-test, * *p* < 0.05, *** *p* < 0.001.

The proximal promoter contains multiple binding sites specific to diverse transcription factors. To identify *Igf2* P3 (−168/+175 relative to the TSS) binding sites related to a decrease in *Igf2* P3 promoter activity, we designed and tested on day 2 P.I. a series of truncation constructs of the *Igf2* P3 promoter vector, with the same transient luciferase reporter system as previously explained (Figure 4A).

In mock- and CV-B4-infected cells (MOI = 0.05), a gradual decrease in the promoter activity was observed with progressive shortening of the *Igf2* P3 promoter, as shown from P291 (−116/+175) to P230 (−55/+175), when compared with the full-length *Igf2* P3 promoter. When cells were transfected with construct P197 (−22/+175), *Igf2* P3 promoter activity was undetectable in both mock- and CV-B4-infected cells. Therefore, the region −168/−22 of *Igf2* P3 is essential for a minimal detection of *Igf2* P3 promoter activity. Additionally, no *Igf2* P3 promoter activity was observed when cells were transfected with *Igf2* P3 promoter constructs containing only regions +6/+175, +26/+175, and +77/+175. Compared with mock-infected cells, the promoter activity of constructs P243 (−68/+175) and P230 (−55/+175) was significantly lower in CV-B4-infected cells (P243, *p* = 0.0468, and P230, *p* = 0.041), whereas no significant difference was detected when transfected with construct P291 (−116/+175) (*p* = 0.0724) (Figure 4B).

Therefore, the *Igf2* P3 promoter region −68 to −22 is targeted and downregulated during CV-B4 infection.

To determine whether *Igf2* P3 promoter region −168/−116 contributes to the decrease in the *Igf2* P3 promoter activity, cells were transfected with construct P248*, containing region −168/−116, and deleted for the region −116/−22. Although region −168 to −116 represents less than 10% of the *Igf2 P3* promoter activity, a significant decrease in the *Igf2* P3 promoter activity with construct P248* was detected in CV-B4-infected cells (*p* = 0.0191). Consequently, this region also appeared to be targeted and downregulated during the CV-B4 infection. When cells were transfected with construct P307 (deleted for the promoter region −151/−116), the *Igf2* P3 promoter activity was higher in both mock- (*p* = 0.0553) and CV-B4-infected cells (*p* = 0.0465) than in the cells transfected with the full *Igf2* P3 promoter (Figure 4C).

### 3.4. CV-B4 Infection Decreases STAT3 Phosphorylation

**Figure 5:**
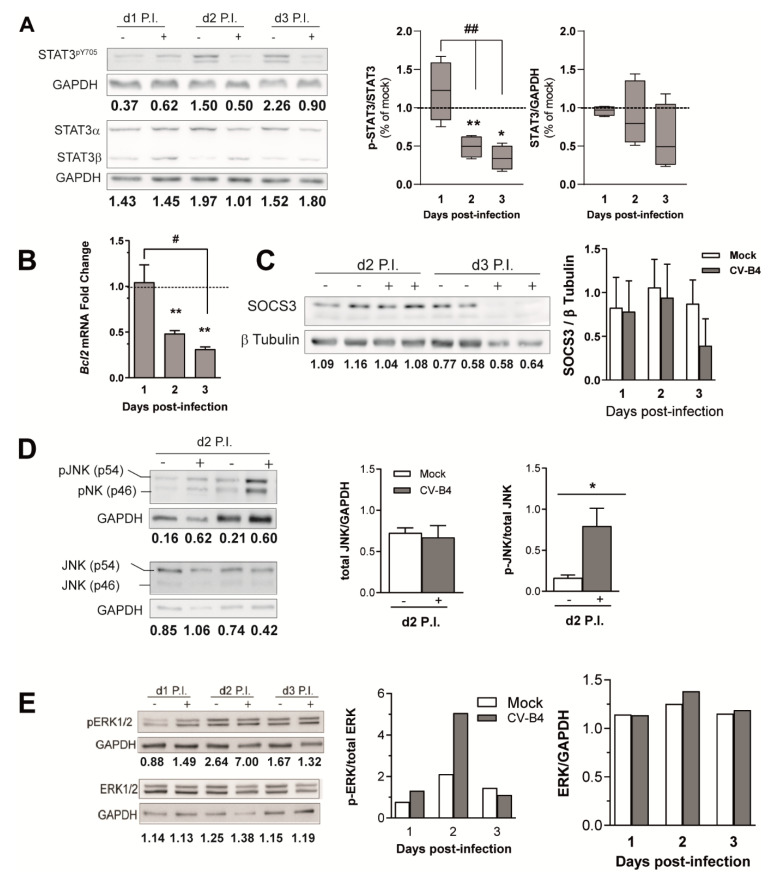
Effect of CV-B4 infection on signal transducer and activator of transcription 3 (STAT3) phosphorylation and on the STAT3 signaling pathway. (**A**) Left panel, Western blot analysis of STAT3^total^ and STAT3^pY705^ in CV-B4-infected cells (+) and mock-infected cells (−); right panel, STAT3^total^ and STAT3^pY705^ relative quantification in CV-B4-infected cells. Box-and-whisker plots extend from minimum to maximum values, with lines at medians; mock samples are represented as a dashed line set at y = 1; *n* = 4–5. (**B**) *Bcl2* mRNA fold change in CV-B4-infected cells (gray histograms) relative to day-matched mock-infected cells; *n* = 3–6. (**C**) Left panel, Western blot analysis of suppressor of cytokine signaling 3 (SOCS3) in CV-B4-infected cells (+) and mock-infected cells (−); right panel, SOCS3 relative quantification; *n* = 2–3. (**D**) Left panel, Western blot analysis of c-Jun *n*-terminal kinase (JNK) ^total^ and phosphorylated JNK in CV-B4-infected cells (+) and mock-infected cells (−); two independent experiments are shown. Right panel, JNK ^total^ and phosphorylated JNK relative quantification in CV-B4-infected cells (+) and mock-infected cells (−); *n* = 3. (**E**) Left panel, Western blot analysis of total extracellular signal-regulated protein kinase (ERK) and phosphorylated ERK in CV-B4-infected cells (+) and mock-infected cells (−). Right panel, ERK^total^ and phosphorylated ERK relative quantification in CV-B4-infected cells (+) and mock-infected cells (−); *n* = 1. (**B**–**E**) The histograms represent average ± SEM. (**A**–**E**), CV-B4-infected cells with multiplicity of infection (MOI) = 0.05. (**A**–**D**) Ratio paired *t*-test, ** *p* < 0.01, and * *p* < 0.05; (**A**,**B**) One-way ANOVA, ## *p* < 0.01 and # *p* < 0.05. Representative Western blots are presented in Supplementary Figure S8A–D.

STAT3 protein expression and phosphorylation (STAT3^pY705^) in MTE4-14 cells were analyzed using Western blot. Although during CV-B4 infections STAT3^pY705^ levels decreased gradually (*p* = 0.0292), STAT3^total^ levels were not significantly different (*p* = 0.3198). The median relative value of STAT3^pY705^ in CV-B4-infected cells was 51% lower on day 2, and 66% lower on day 3 P.I. than in mock-infected cells (Figure 5A). Similarly, *Bcl2*, a STAT3 response gene [39], was downregulated during the CV-B4 infection of MTE4-14 cells (*p* = 0.0304) at an average of 52% on day 2 and 69% on day 3 (Figure 5B). STAT3^pY705^ levels increased in mock-infected cells between days 1 and 3 P.I. (Figure 5A and Supplementary Figure S5A). Accordingly, an increase in *Bcl2* (B-cell lymphoma 2 ) mRNA expression was observed in mock-infected cells; however, *P53* mRNA expression, an irrelevant mRNA for STAT3^pY705^ signaling, did not increase in mock-infected cells between days 1 day and 3 P.I. (Supplementary Figure S5B,C).

An analysis of regions −68/−22 and −168/−116 using a bioinformatic prediction tool [40] led us to identify an E2F binding site in the −168/−116 promoter region (Supplementary Table S2). Interestingly, STAT3 can recognize the E2F binding site [41]. In addition, a construct containing three sequential binding sites for E2F (E2FX3) was developed. Although these additional binding sites for E2F resulted in a higher *Igf2* P3 promoter activity in mock-infected cells (*p* = 0.0378) than that obtained using the full *Igf2* P3 promoter, these additional binding sites had no effect in CV-B4-infected cells (*p* = 0.2111) (Supplementary Figure S6).

Classical members of the STAT3^pY705^ activation pathway were also analyzed. Although we detected an increase in *Il6* (a well-known STAT3^pY705^ activator) in CV-B4-infected cells (Supplementary Figure S7A), we did not detect any significant difference between mock- and CV-B4-infected cells for the receptor IL6Rα (CD126), analyzed using flow cytometry (Supplementary Figure S7B). The protein expression of suppressor of cytokine signaling 3 (SOCS3), a negative regulator of the classical JAK/STAT3 pathway, was also investigated using Western blot. However, no upregulation was observed during the CV-B4 infection (Figure 5C). Owing to their ability to downregulate STAT3^pY705^ via the activation of STAT3^pS727^ [42], c-Jun *N*-terminal kinase (JNK) [43] and extracellular signal-regulated protein kinase (ERK) protein levels [44] were also analyzed in mock- and CV-B4-infected cells. Two days P.I., both the phosphorylated JNK and phosphorylated ERK levels increased, whereas JNK ^total^ or ERK ^total^ levels remains unchanged (Figure 5D,E). These results suggest a possible role of these kinases in STAT3^pY705^ downregulation during CV-B4 infection of MTE4-14 cells.

### 3.5. Decrease in Igf2 Transcripts and Downregulation of Pro-IGF2 Expression in TECs Following CV-B4 Inoculation In Vivo

To investigate the CV-B4 effect on *Igf2* transcript isoforms in TECs in an in vivo model, outbred Swiss mice were intraperitoneally inoculated with CV-B4. Nonetheless, the TECs, where IGF2 is localized [1], are rare and represent less than 1% of the thymus cellularity (with 99% are thymocytes or immature T-cells). Owing to this, we performed enzymatic digestion of the thymus, followed by depletion of CD45-positive thymocytes from digested thymic cells to enrich the TECs (CD45-negative) subpopulation (Supplementary Figure S9A). TECs (CD45—EpCAM+) were enriched 62-fold in CD45-negative sorted cells (Supplementary Figure S9B), and accordingly, the mean *total Igf2* relative mRNA level was 318-fold higher in CD45-sorted cells (TECs) than in total thymic cells (not depleted) (Supplementary Figure S9C).

**Figure 6:**
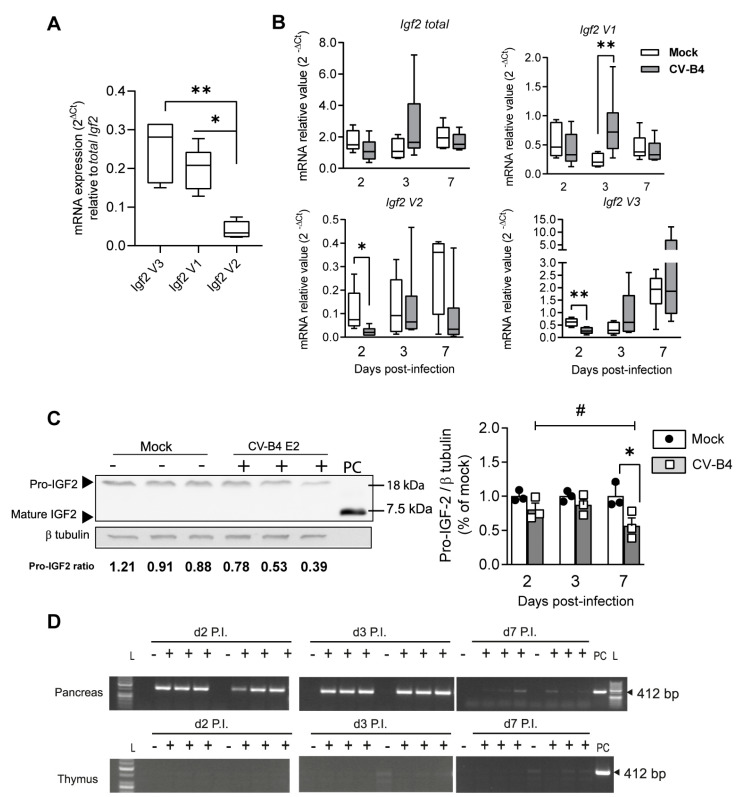
Thymic Igf2 expression following CV-B4 inoculation in vivo. (**A**) Relative mRNA expression of *Igf2 V3*, *V1*, and *V2* mRNA transcript isoforms in Cluster Differentiation 45 (CD45)-negative enriched thymic epithelial cells (TECs) in mock-inoculated mice on day 3 P.I.; *n* = 6. (**B**) Relative mRNA expression of the *Igf2* mRNA transcript isoform in mock- and CV-B4-inoculated mice; box-and-whisker plots extend from minimum to maximum values with lines indicating medians; *n* = 5–6. Raw Ct values are indicated in Supplementary Table S1. (**C**) Left panel, representative Western blot of IGF2 and its precursors on day 7 P.I. in mock- (−) and CV-B4-inoculated mice (+). Independent biological samples are represented. PC, purified mature IGF2. Representative full-length Western blots of IGF2 and β-tubulin are presented in Supplementary Figure S9E. Right panel, relative quantification of pro-IGF2 in mock- and CV-B4-inoculated mice; the histogram represents the mean of relative value ± SD; *n* = 3. For relative quantification of pro-IGF2 expression, the ratios on days 2, 3, or 7 are normalized to the mean ratio of pro-IGF2 of mock samples on days 2, 3, or 7, respectively. (**D**) Agarose gel electrophoresis of one-step RT-PCR products of the CV-B4 genome in the digested thymus (total thymic cells) and matched pancreas in CV-B4- (+) and mock-inoculated mice (−). Independent biological samples are shown. L, ladder; PC, MTE4-14. Uncropped gels are presented in Supplementary Figure S9F. (**A**) One-way ANOVA, * *p* < 0.05, and ** *p* < 0.01. (**B**,**C**) Student’s *t*-test, * *p* < 0.05, and ** *p* < 0.01. (**C**) One-way ANOVA, # *p* < 0.05.

First, the expression of *Igf2* transcript isoforms of mock-infected mice were determined in enriched TECs using RT-qPCR (Figure 6A). Among *Igf2* transcripts, *Igf2 V1*, *V2*, and *V3* were found in enriched TECs, with *Igf2 V3* mRNA as the main *Igf2* isoform and *Igf2 V2* mRNA as the minor *Igf2* isoform (Figure 6A). Second, the median relative values of *Igf2* isoforms in mock- and CV-B4-inoculated mice were compared. Results indicate that *Igf2 V3* and *V2* decreased after 2 days P.I. *Igf2 V3* was 58% lower (*p* = 0.0096) and *Igf2 V2* was 72% lower (*p* = 0.0157) in CV-B4-inoculated mice than in mock-infected mice (Figure 6B).

However, in CV-B4-inoculated mice, *Igf2 Variant 1* (*V1*) was upregulated at 257% (*p* = 0.0047) at 3 days P.I. and *total Igf2* level (all *Igf2* transcripts) in TECs was unchanged. Seven days P.I., the relative expression of each isoform was comparable with that in mock-inoculated mice (Figure 6B). Of note, while outlier sample value of *Igf2* expression (*Igf2 total*, *Igf2 V3,* and *Igf2 V1* as well) was noticed among CV-B4-infected mice on day 3 P.I. (Supplementary Table S1), no significant difference for *Hprt* cycle threshold (Ct) is found between mock and CV-B4-infected mice (Supplementary Figure S10), which suggest an intragroup variability. An individual intervariability was observed as far as *Igf2* Ct values (*Igf2 total*, *Igf2 V3*, and *Igf2 V1* as well) are concerned in CV-B4-infected mice on day 3 P.I. (Supplementary Table S1). Indeed, in one of the animals, the *Igf2* Ct (Cycle Threshold) values were lower than those in the rest, whereas there was no significant difference in *Hprt* Ct between mock and CV-B4-infected mice (Supplementary Figure S10).

We then analyzed the IGF2 protein level and its precursors in the total thymic cell population (containing immature T-cells and TECs) using Western blot. Though no mature IGF2 was found, we detected the pro-IGF2 precursor form (18 kDa, dominant isoform). The pro-IGF2 level on day 7 P.I. was 43% lower (*p* = 0.0372) in CV-B4-inoculated mice than in mock-inoculated mice (Figure 6C). We noticed that the TECs percentage in CV-B4-inoculated mice remains unchanged compared with mock-inoculated mice (Supplementary Figure S9D). Collectively, these results suggest that the CV-B4 infection can modulate Igf2 expression at the mRNA and protein levels in the thymus.

CV-B4 RNA was detected in total thymic cells (Supplementary Figure S9A), owing to the limiting RNA amount isolated from CD45-negative TECs enriched samples. Although CV-B4 RNA was detected in all pancreatic tissues harvested from CV-B4-inoculated mice, it was absent in all total thymic cell samples of the CV-B4-inoculated mice (after enzymatic digestion) (Figure 6D).

## 4. Discussion

We took advantage of the MTE4-14 cell line to investigate the effect of the CV-B4 infection on the *Igf2* expression. In this model, the *total Igf2* mRNA level was decreased following CV-B4 infection. Among *Igf2* mRNA transcripts, *Igf2 V3* and *Igf2 V1*, the major and minor transcripts, respectively, were strongly downregulated during the CV-B4 infection. It has been observed that the decrease in the *Igf2 V3* major isoform was owing to a decrease in the *Igf2* P3 core promoter activity (−168/+175 relative to the TSS), resulting in *Igf2 V3* downregulation at the transcriptional level. Moreover, the mRNA stability of *Igf2 V3* was unchanged in infected cells, which confirm the regulation of *Igf2 V3* mRNA at the transcriptional level. In addition, previous studies have indicated high stability of *total Igf2* mRNA with similar mRNA half-life values [45,46]. As, *Igf2* transcripts have high stability, we expect *Igf2 V1* to have high stability and consequently, to be affected similarly than *Igf2 V3*, at the transcriptional level. Further analysis of *Igf2* P2 promoter activity at later time points (3 days P.I.) is required to confirm this hypothesis.

Our results also indicate that the two promoter regions of *Igf2* P3, −68/−22 and −168/−116 (both relative to the TSS), were negatively regulated during the CV-B4 infection. A predictive analysis of *Igf2* P3 −68/−22 was performed and indicates putative binding sites for SPs/KLFs’ transcription factors. Even though SP1 was accordingly identified as a positive activator of human *IGF2 P4* (homologous to murine *Igf2* P3 promoter), the KLF transcription factor is essential for thymocytes’ development and differentiation [47,48]. Future studies with point mutants and binding assays are, nonetheless, required to validate these binding sites to conclude their role in IGF2 regulation in the context of CV-B4 infections.

In the MTE4-14 cell line, *Igf2* downregulation occurred concomitantly with CV-B4 genome detection within the cells and CV-B4 production in the supernatant. Clearly, MTE4-14 cells were infected with the virus, which is in agreement with the detection by RT-PCR in these cells of the Coxsackievirus and adenovirus receptor (CAR), required by CV-B4 for entry into host cells (Supplementary Figure S11). Interestingly, no IGF2 downregulation was reported when MTE4-14 cells were inoculated with supernatants from UV light-inactivated CV-B4-infected cells [18].

Although picornaviruses replicate exclusively in the cytoplasm of infected cells, it is surprising that in our model, they can affect host cell transcription of *Igf2*. It has been reported that enteroviral proteases 3C and 2A can cleave eukaryotic transcription factors or transcriptional activators. Moreover, Picornavirus and Enterovirus proteases were described to hijack nucleocytoplasmic trafficking [49]. It cannot be excluded that some host transcription factors are cleaved or cannot shuttle to the nuclei, resulting in the alteration of cellular transcription.

The transcription factor STAT3 essential for TECs development and survival and contributing to Igf2 expression was investigated. In this study, we observed that CV-B4 infection induces a decrease in STAT3pY705 in MTE4-14 cells.

Our analysis of the *Igf2* P3 region −168/−116 (related to the TSS) revealed an E2F transcription factor binding site, recognizable by STAT3 [41]. Of note, the E2F transcription factor, regulates genes, which play a role in cell proliferation [50]. We realized a construct with three adjacent binding sites for E2F transcription factor, which would stimulate the basal *Igf2* P3 promoter activity. Indeed, the E2FX3 *Igf2* P3 promoter activity was increased only in mock-infected cells, contrary to CV-B4-infected cells, for which no improvement of *Igf2* P3 was observed. In mock-infected cells, contrary to the CV-B4-infected cells, STAT3pY705 could have effectively bound to more E2F binding sites than with the full *Igf2* P3 promoter (comprising one E2F binding sites), thus stimulating *Igf2* P3 promoter activity.

Another striking element connecting STAT3pY705 to *Igf2 V3* in our model is the parallel increase in STAT3pY705 and *Igf2 V3* in mock-infected cells during culture. Thus, the decrease in *Igf2* P3 promoter activity might be related, at least in part, to the decrease in STAT3pY705.

As previously reported in MTE4-14 cells and TECs, IL-6 expression increased during the CV-B4 infection [16,18]. IL-6, not only an inflammatory marker but also a well-known inducer of STAT3pY705 phosphorylation, increased during CV-B4 infection. IL-6Rα (CD126) and the inhibitor of IL-6 signaling SOCS3 [51] were analyzed, but their respective levels remained unaffected during the CV-B4 infection. JNK and ERK are MAP kinase members contributing to CV-Bs’ viral progeny release [52,53,54] and STAT3pY705 downregulation [43,44]. Their phosphorylation levels increased during the CV-B4 infection, suggesting that viral upregulation of phosphorylated JNK and ERK also contributes to the decrease in STAT3pY705 and IGF2 in MTE4-14 cells infected with CV-B4. Further experiments with respective inhibition of these kinases will confirm this hypothesis.

Notably, the decrease in IGF2 has been reported to be not an exclusive effect of CV-B4 E2 in this cell line as other strains of Enterovirus B, such as CV-B3 Nancy and CV-B4 JVB, can also decrease the level of (pro) IGF2 in MTE4-14 cells [18]. Thus, not only CV-B4 but also other coxsackieviruses-B could alter STAT3 signaling and *Igf2* transcriptional activity, potentially leading to the loss of development of central tolerance to insulin.

In this study, we investigated the expression of *Igf2* transcripts in primary TECs obtained from Swiss albino mice infected with CV-B4. It was reported that these mice can be successfully infected with CV-B4 [13,55]. As *Igf2* mainly localizes in TECs, a rare cell thymic subtype, a protocol using depletion of thymocytes and TECs enrichment was required.

In mock-inoculated mice, we found *Igf2 V3* and *Igf2 V1* as isoforms mainly in CD45-negative enriched TECs, which corroborate with previous findings on human TECs showing that *IGF2* P3 and *IGF*2 P4, orthologous promoters for murine *Igf2 V1* and *Igf2 V3*, respectively, are active in these cells [56]. Further, fewer *Igf2 V2* (orthologous murine transcript for human *IGF2 P2*) transcripts were found in CD45-negative enriched TECs. In CV-B4-inoculated mice, we observed a decrease in *Igf2 V3* and *Igf2 V2*. Although an increase in *Igf2 V1* mRNAs was observed in that model, a decrease in both *Igf2 V3* and *Igf2 V2* was sufficient to achieve a decrease in the pro-IGF2 protein level in CV-B4-infected mice. Thus, this observed downregulation of *Igf2 V3* and pro-IGF2 has the potential to alter the development of self-immune tolerance to insulin, as IGF2 expression is required for the complete immune self-tolerance to insulin [3] and the differentiation of precursor T-cells [56,57]. We noticed the absence of mature IGF2 forms in the murine thymus. A differential processing of IGF2 may occur in the thymus as in aged rat brains, where no mature IGF2 is detected [58]. Indeed, a reduced expression or activity of the enzyme cleaving pro-IGF2 to mature IGF2 can reduce IGF2 processing and detection of mature IGF2 [58,59].

In our experiments CV-B4-inoculated Swiss mice were effectively infected, as proved by CV-B4 RNA detection in their pancreas. Contrastingly, CV-B4 RNA was not detected in unsorted total thymic cells isolated after enzymatic digestion, which is part of the protocol adapted for *Igf2* quantification in CD45-negative enriched TECs. It cannot be excluded that this protocol restricts CV-B4 RNA detection within thymic cells and/or that the viral load in TECs in vivo is under the limit of detection of the RT-PCR.

Whether TECs can be infected with CV-B4 in vivo remains to be determined, nevertheless, a downregulation of IGF2 expression in TECs of mice infected with the virus has been observed, which raises the hypothesis of indirect mechanisms involved in this effect. It was reported that both IFN-α and -β are inhibitors of IGF2 expression [60,61,62]. Besides, it was shown that inactivated CV-B virions can upregulate IFN production in immune cells, revealing that active intracellular viral replication is not required for IFN induction [63,64,65]. Thus, it is possible that in Swiss mice inoculated with CV-B4, mediators such as IFNs produced by various cells (e.g., thymocytes, monocytes, and TECs) may modulate the IGF2 expression by TECs. Further studies are needed to address this issue.

Overall, this study showed that CV-B4 infection can modulate the expression of IGF2 in a model of TECs in vitro and can downregulate the *Igf2 V3* expression and protein synthesis in primary TECs in vivo.

## 5. Conclusions

**Figure 7:**
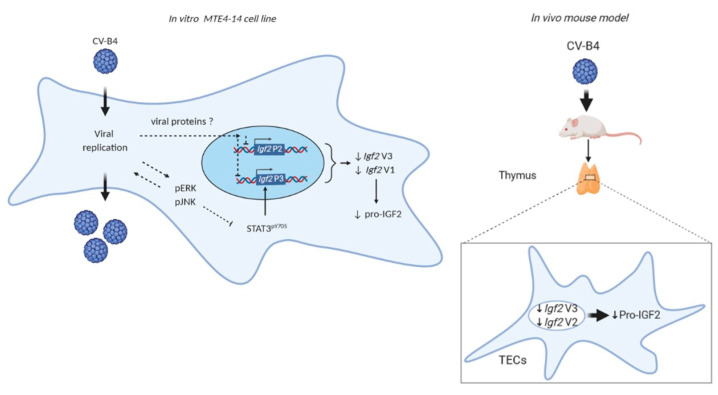
The effect of CV-B4 infection on *Igf2* expression in vitro and in vivo.

The main discoveries of this study are the following: first, in the thymic epithelial MTE4-14 cell line, CV-B4 infection decreased the transcriptional activity of the murine *Igf2* P3 promoter together with *Igf2 V3* and *V1* mRNA, which resulted in the decrease in the pro-IGF2 level. We also reported a parallel decrease in STAT3 phosphorylation and the activation of ERK and JNK kinases. In that model, CV-B4 can replicate and complete the viral cycle, thus a direct role of CV-B4 in Igf2 downregulation is conceivable. Second, in mice inoculated intraperitoneally with CV-B4, the *Igf2 V3* mRNA-dominant transcript temporarily decreased in TECs and resulted also in a decrease in pro-IGF2 protein (Figure 7). In conclusion, these findings shed new light on the thymic IGF2 regulation by CV-B4 infection, thus strengthening the hypothesis of a possible role of CV-B infections in decreasing central tolerance to insulin.

## Figures and Tables

**Table 1:** Primers list for RT-qPCR and for site-directed mutagenesis.

	RT-qPCR Primers	
Gene	Sequence 5′-3′	PCR Product Size (bp)
*Hprt*	Forward: TTATCAGACTGAAGAGCTACTGTAATG Reverse: CTTCAACAATCAAGACATTCTTTCC	108
*Total Igf2*	Forward: GGGAGCTTGTGGACACGC Reverse: GCACTCTTCCACGATGCCA	107
*Igf2 V1*	Forward: CCGGCTTCCAGGTACCAAT Reverse: GCAGCGATGCAGCACAAG	91
*Igf2 V2*	Forward: GCCCTTCTCCTCCGATCCT Reverse: ATGAGAAGCACCAACATCGACTT	99
*Igf2 V3*	Forward: CCAGCCTTTTCCTGTCTTCATC reverse: CCATTGGTACCTGAAGTTGGGTAA	69
*Birc5*	Forward: TCTGGCAGCTGTACCTCAAGAACT Reverse: AAACACTGGGCCAAATCAGGCT	148
*Tp53*	Forward: TTCATTGGGACCATCCTGGC Reverse: TGGCAGTCATCCAGTCTTCG	121
*Bcl2*	Forward: GTGAACTGGGGGAGGATTGT Reverse: GGAGAAATCAAACAGAGGCC	216
*Il6*	Forward: GTTCTCTGGGAAATCGTGGA Reverse: TGTACTCCAGGTAGCTATGG	208
*Cxadr*	Forward: GTCTAGTCGCAGCATACAC	287
	Reverse: TTCCTGCTGACCGTTCTTG	
*Aire*	Forward: GGGACTGGTTTAGGTCCACA	326
	Reverse: AGGTGGGGATGGAATGCTAC	
*Beta actin*	Forward: ATGCTCCCCGGGCTGTAT	87
	Reverse: CATAGGAGTCCTTCTGACCCATTC	
	**Site-directed mutagenesis primers**	
***Igf2* P3 Plasmid** **(Sequence Relative to TSS)**	**Primer**	**Annealing (°C)**
P243 (−68/+175)	Forward: GGTAGGGTGGAGCCGGGA Reverse: GAGCTCAGGTACCGGCCA	68
P149 (+26/+175)	Forward: AACCTTCCAGCCTTTTCCTGT Reverse: GAGCTCAGGTACCGGCCA	66
P98 (+77/+175)	Forward: TTACCCAACTTCAGGTAACCAGG Reverse: GAGCTCAGGTACCGGCCA	70
P197 (−22/+175)	Forward: GGAGGCACTGACCAGTTCG Reverse: GAGCTCAGGTACCGGCCA	67
P169 (+6/+175)	Forward: ACATTAGCTTCTCCTGTGAGAACC Reverse: GAGCTCAGGTACCGGCCA	65
P291 (−116/+175)	Forward: GCGGGTGCAAAGGGGGCG Reverse: GAGCTCAGGTACCGGCCAGTTAG	70
P230 (−55/+175)	Forward: GGGACTGGGAGGAGCCAC Reverse: GAGCTCAGGTACCGGCCA	71
P248 * (−167/−116^Δ^^−^^116/−^^22^−22/+175)	Forward: CGGAGGCACTGACCAGTTC Reverse: CCCCTCCCTCCTTCCAGC	67
P307 * (−167/−151^Δ^^−^^151/−^^116^−116/+175)	Forward: GGCGGGTGCAAAGGGGGC Reverse: CACCCCCAAGAGCTAGCGAGC	69
E2FX3	Forward: GGAAGTGGCTGGAAGTGGCTGGAAGGAGGGAGG	66
Sequencing primer	Forward: CTAGCAAAATAGGCTGTCCC Reverse: ACTGCATTCTAGTTGTGGTTTGC	

*, represents a promoter construct partly deleted for a nucleotide sequence indicated by the symbol Δ.

**Table 2:** List of antibodies used for Western blot.

Primary Antibody	Dilution	Supplier	Clone
Rabbit anti-IGF2	1:500	AVIVA SYSTEMS BIOLOGY	OAAB07463
Rabbit anti-STAT3	1:1000	Cell Signaling Technology	D3Z2G
Rabbit anti-STAT3 (p705 Tyr)	1:1000	Cell Signaling Technology	D3A7
Rabbit anti-JNK/SAPK	1:1000	Cell Signaling Technology	
Rabbit anti-pJNK	1:1000	Cell Signaling Technology	81E11
Rabbit anti-ERK (1/2)	1:1000	Cell Signaling Technology	137F5
Rabbit anti-pERK (1/2)	1:1000	Cell Signaling Technology	Antirabbit IgG HRP
Rabbit anti-SOCS3	1:1000	Cell Signaling Technology	Antirabbit IgG HRP
Mouse anti-GAPDH	1:1000	Pierce	GA1R
Mouse anti-β tubulin	1:1000	Pierce	BT7R
Mouse anti-VP1	1:1000	Dako	5-D8/1

## Data Availability

Data is contained within the article or supplementary material.

## Supplementary Materials

The following are available online at https://www.mdpi.com/2076-2607/9/2/402/s1, Figure S1: Microscopic examination of mock- and Coxsackievirus B4 (CV-B4)-infected MTE4-14 cells 1–3 days postinfection (P.I.), Figure S2A: Primer localization in insulin-like growth factor 2 (Igf2) mRNA transcripts, Figure S2B: Uncropped whole PCR gel for Figure 1A, Figure S3A: Relative mRNA expression of the *total Igf2* mRNA in CV-B4-infected cells (multiplicity of infection (MOI) = 0.005 to MOI = 0.5) 2 days P.I., Figure S3B: Relative mRNA expression of *Igf2 V3* and *total Igf2* mRNA in mock-infected MTE4-14 cells 1, 2, and 3 days P.I., Figure S3C: 3-(4,5-Dimethyl-2-thiazolyl)-2,5-diphenyl-2H-tetrazolium bromide reagent (MTT) cell viability assay analysis on day 2 and 3 P.I., Figure S3D: Full-length Western blot for Figure 2C, Figure S3E: Uncropped whole PCR gel for Figure 2D, Figure S3F: Full-length Western blot for Figure 2E, Figure S4: Effect of CV-B4 on *Igf2* transcript stability, Figure S5: Parallel increase in signal transducer and activator of transcription 3 (STAT3)^pY705^ with *Bcl2* in mock cells, Figure S6: Effect of CV-B4 on *Igf2* P3 promoter with the E2FX3 construct, Figure S7: *Il6* mRNA relative expression and flow cytometry analysis of extracellular IL-6Rα in mock- (−) or in CV-B4-infected cells (+), Figure S8A: Full-length Western blot for Figure 5A, Figure S8B: Full-length Western blot for Figure 5C, Figure S8C: Full-length Western blot for Figure 5D, Figure S8D: Full-length Western blot for Figure 5E, Figure S9A: Thymus processing steps for isolation of total thymic cells (unsorted cells) and CD45-negative enriched cells (thymic epithelial cells (TECs)), Figure S9B: Flow cytometry analysis of CD45-negative enriched TECs (EpCAM+CD45−) versus the unsorted total thymic population, Figure S9C: Relative mRNA expression of the *total Igf2* mRNA in the unsorted total thymic population and matched enriched TECs fraction (CD45−), Figure S9D: Flow cytometry analysis of total thymic cells 2, 3, and 7 days P.I., Figure S9E: Full-length Western blot for Figure 6C, Figure S9F: Uncropped whole PCR gels for Figure 6D, Figure S10: *Hprt cycle threshold* in mice (A) and in MTE4-14 cells (B), Figure S11: Coxsackievirus and adenovirus receptor (CAR) mRNA expression in MTE4-14 cells, Supplementary Table S1: Raw Ct of *Igf2* expression in Figure 6B, Supplementary Table S2: Putative transcription factor binding sites in regions −68/−22 and −168/−116.

## References

[1] Geenen V., Trussart C., Michaux H., Halouani A., Jaïdane H., Collée C., Renard C., Daukandt M., Ledent P., Martens H. (2019). The presentation of neuroendocrine self-peptides in the thymus: An essential event for individual life and vertebrate survival. Ann. N. Y. Acad. Sci..

[2] Geenen V., Louis C., Martens H., Registry T.B.D. (2004). An Insulin-like Growth Factor 2-Derived Self-Antigen Inducing a Regulatory Cytokine Profile after Presentation to Peripheral Blood Mononuclear Cells from DQ8+Type 1 Diabetic Adolescents: Preliminary Design of a Thymus-Based Tolerogenic Self-Vaccination. Ann. N. Y. Acad. Sci..

[3] Hansenne I., Renard-Charlet C., Greimers R., Geenen V. (2006). Dendritic Cell Differentiation and Immune Tolerance to Insulin-Related Peptides in Igf2-Deficient Mice. J. Immunol..

[4] Shapiro M.R., Wasserfall C.H., McGrail S.M., Posgai A.L., Bacher R., Muir A., Haller M.J., Schatz D.A., Wesley J.D., Von Herrath M. (2020). Insulin-Like Growth Factor Dysregulation Both Preceding and Following Type 1 Diabetes Diagnosis. Diabetes.

[5] Yang G., Geng X.-R., Song J.-P., Wu Y., Yan H., Zhan Z., Yang L., He W., Liu Z.-Q., Qiu S. (2014). Insulin-like growth factor 2 enhances regulatory T-cell functions and suppresses food allergy in an experimental model. J. Allergy Clin. Immunol..

[6] Geng X.-R., Yang G., Li M., Song J.-P., Liu Z.-Q., Qiu S., Liu Z., Yang P.-C. (2014). Insulin-like Growth Factor-2 Enhances Functions of Antigen (Ag)-specific Regulatory B Cells. J. Biol. Chem..

[7] Kecha-Kamoun O., Achour I., Martens H. (2001). Thymic expression of insulin-related genes in an animal model of autoimmune type 1 diabetes. Diabetes Metab. Res. Rev..

[8] Hober D., Sauter P. (2010). Pathogenesis of type 1 diabetes mellitus: Interplay between enterovirus and host. Nat. Rev. Endocrinol..

[9] Yoon J.W., Austin M., Onodera T., Notkins A.L. (1979). Virus-Induced Diabetes Mellitus: Isolation of a Virus from the Pancreas of a Child with Diabetic Ketoacidosis. N. Engl. J. Med..

[10] Vargová A., Bopegamage S., Borsanyiová M., Petrovičová A., Benkovičová M. (2003). Coxsackievirus infection of mice. II. Viral kinetics and histopathological changes in mice experimentally infected with Coxsackievirus B3 by intraperitoneal route. Acta Virol..

[11] Bopegamage S., Kovacova J., Vargova A., Motusova J., Petrovicova A., Benkovicova M., Gomolcak P., Bakkers J., Van Kuppeveld F., Melchers W.J.G. (2005). Coxsackie B virus infection of mice: Inoculation by the oral route protects the pancreas from damage, but not from infection. J. Gen. Virol..

[12] Chatterjee N.K., Hou J., Dockstader P., Charbonneau T. (1992). Coxsackievirus B4 infection alters thymic, splenic, and peripheral lymphocyte repertoire preceding onset of hyperglycemia in mice. J. Med Virol..

[13] Jaïdane H., Gharbi J., Lobert P.-E., Lucas B., Hiar R., Ben M’Hadheb M., Brilot F., Geenen V., Aouni M., Hober D. (2006). Prolonged Viral RNA Detection in Blood and Lymphoid Tissues fromCoxsackievirus B4 E2Orally-InoculatedSwissMice. Microbiol. Immunol..

[14] Brilot F., Geenen V., Hober D., Stoddart C.A. (2004). Coxsackievirus B4 Infection of Human Fetal Thymus Cells. J. Virol..

[15] Brilot F., Jaïdane H., Geenen V., Hober D. (2008). Coxsackievirus B4 infection of murine foetal thymus organ cultures. J. Med. Virol..

[16] Brilot F., Chehadeh W., Charlet-Renard C., Martens H., Geenen V., Hober D. (2002). Persistent Infection of Human Thymic Epithelial Cells by Coxsackievirus B4. J. Virol..

[17] Lepesant H., Pierres M., Naquet P. (1995). Deficient Antigen Presentation by Thymic Epithelial Cells Reveals Differential Induction of T Cell Clone Effector Functions by CD28-Mediated Costimulation. Cell. Immunol..

[18] Jaïdane H., Caloone D., Lobert P.-E., Sane F., Dardenne O., Naquet P., Gharbi J., Aouni M., Geenen V., Hober D. (2012). Persistent Infection of Thymic Epithelial Cells with Coxsackievirus B4 Results in Decreased Expression of Type 2 Insulin-Like Growth Factor. J. Virol..

[19] Bergman D., Halje M., Nordin M., Engström W. (2013). Insulin-Like Growth Factor 2 in Development and Disease: A Mini-Review. Gerontology.

[20] Panda A.C., Grammatikakis I., Yoon J.-H., Abdelmohsen K. (2013). Posttranscriptional Regulation of Insulin Family Ligands and Receptors. Int. J. Mol. Sci..

[21] Liu M., Roth A., Yu M., Morris R., Bersani F., Rivera M.N., Lu J., Shioda T., Vasudevan S., Ramaswamy S. (2013). The IGF2 intronic miR-483 selectively enhances transcription from IGF2 fetal promoters and enhances tumorigenesis. Genes Dev..

[22] Lui J.C., Baron J. (2013). Evidence that Igf2 down-regulation in postnatal tissues and up-regulation in malignancies is driven by transcription factor E2f3. Proc. Natl. Acad. Sci. USA.

[23] Wang K., Wang C., Xiao F., Wang H., Wu Z. (2008). JAK2/STAT2/STAT3 are required for myogenic differentiation. J. Biol. Chem..

[24] Lee S.-C., Min H.-Y., Jung H.J., Park K.H., Hyun S.Y., Cho J., Woo J.K., Kwon S.J., Lee H.-J., Johnson F.M. (2016). Essential role of insulin-like growth factor 2 in resistance to histone deacetylase inhibitors. Oncogene.

[25] Himes B.E., Obraztsova K., Lian L., Shumyatcher M., Rue R., Atochina-Vasserman E.N., Hur S.K., Bartolomei M.S., Evans J.F., Krymskaya V.P. (2018). Rapamycin-independent IGF2 expression in Tsc2-null mouse embryo fibroblasts and human lymphangioleiomyomatosis cells. PLoS ONE.

[26] Satoh R., Kakugawa K., Yasuda T., Yoshìda H., Sibilia M., Katsura Y., Levi B., Abramson J., Koseki Y., Koseki H. (2016). Requirement of Stat3 Signaling in the Postnatal Development of Thymic Medullary Epithelial Cells. PLoS Genet..

[27] Lomada D., Jain M., Bolner M., Reeh K.A.G., Kang R., Reddy M.C., DiGiovanni J., Richie E.R. (2016). Stat3 Signaling Promotes Survival And Maintenance Of Medullary Thymic Epithelial Cells. PLoS Genet..

[28] Sano S., Takahama Y., Sugawara T., Kosaka H., Itami S., Yoshikawa K., Miyazaki J.-I., Van Ewijk W., Takeda J. (2001). Stat3 in Thymic Epithelial Cells Is Essential for Postnatal Maintenance of Thymic Architecture and Thymocyte Survival. Immunity.

[29] Cheng F., Wang H.-W., Cuenca A., Huang M., Ghansah T., Brayer J., Kerr W.G., Takeda K., Akira S., Schoenberger S.P. (2003). A Critical Role for Stat3 Signaling in Immune Tolerance. Immunity.

[30] Xing Y., Hogquist K.A. (2014). Isolation, identification, and purification of murine thymic epithelial cells. J. Vis. Exp..

[31] Roberts N.A., Adams B.D., McCarthy N.I., Tooze R.M., Parnell S.M., Anderson G., Kaech S.M., Horsley V. (2017). Prdm1 Regulates Thymic Epithelial Function To Prevent Autoimmunity. J. Immunol..

[32] Pourianfar H.R., Javadi A., Grollo L. (2012). A Colorimetric-Based Accurate Method for the Determination of Enterovirus 71 Titer. Indian J. Virol..

[33] Rotwein P., Hall L.J. (1990). Evolution of Insulin-Like Growth Factor II: Characterization of the Mouse IGF-II Gene and Identification of Two Pseudo-Exons. DNA Cell Biol..

[34] Caricasole A., Ward A. (1993). Transactivation of mouse insulin-like growth factor II (IGF-II) gene promoters by the AP-1 complex. Nucleic Acids Res..

[35] Dreos R., Ambrosini G., Périer R.C., Bucher P. (2012). EPD and EPDnew, high-quality promoter resources in the next-generation sequencing era. Nucleic Acids Res..

[36] Bok K., Prikhodko V.G., Green K.Y., Sosnovtsev S.V. (2009). Apoptosis in Murine Norovirus-Infected RAW264.7 Cells Is Associated with Downregulation of Survivin. J. Virol..

[37] Chau D.H.W., Yuan J., Zhang H., Cheung P., Lim T., Liu Z., Sall A., Yang D. (2007). Coxsackievirus B3 proteases 2A and 3C induce apoptotic cell death through mitochondrial injury and cleavage of eIF4GI but not DAP5/p97/NAT1. Apoptosis.

[38] Wang Y., Zhao S., Chen Y., Wang T., Dong C., Wo X., Zhang J., Dong Y., Xu W., Feng X. (2019). The Capsid Protein VP1 of Coxsackievirus B Induces Cell Cycle Arrest by Up-Regulating Heat Shock Protein 70. Front. Microbiol..

[39] Durant L., Watford W.T., Ramos H.L., Laurence A., Vahedi G., Wei L., Takahashi H., Sun H.-W., Kanno Y., Powrie F. (2010). Diverse Targets of the Transcription Factor STAT3 Contribute to T Cell Pathogenicity and Homeostasis. Immunity.

[40] Weirauch M.T., Yang A., Albu M., Cote A.G., Montenegro-Montero A., Drewe P., Najafabadi H.S., Lambert S.A., Mann I., Cook K. (2014). Determination and Inference of Eukaryotic Transcription Factor Sequence Specificity. Cell.

[41] Kiuchi N., Nakajima K., Ichiba M., Fukada T., Narimatsu M., Mizuno K., Hibi M., Hirano T. (1999). STAT3 Is Required for the gp130-mediated Full Activation of the c-myc Gene. J. Exp. Med..

[42] Wakahara R., Kunimoto H., Tanino K., Kojima H., Inoue A., Shintaku H., Nakajima K. (2012). Phospho-Ser727 of STAT3 regulates STAT3 activity by enhancing dephosphorylation of phospho-Tyr705 largely through TC45. Genes Cells.

[43] Gkouveris I., Nikitakis N., Karanikou M., Rassidakis G., Sklavounou A. (2016). JNK1/2 expression and modulation of STAT3 signaling in oral cancer. Oncol. Lett..

[44] Gkouveris I., Nikitakis N., Karanikou M., Rassidakis G., Sklavounou A. (2014). Erk1/2 activation and modulation of STAT3 signaling in oral cancer. Oncol. Rep..

[45] Asfari M., De W., Nöel M., Holthuizen P.E., Czernichow P. (1995). Insulin-like growth factor-II gene expression in a rat insulin-producing beta-cell line (INS-1) is regulated by glucose. Diabetologia.

[46] Erbay E., Park I.-H., Nuzzi P.D., Schoenherr C.J., Chen J. (2003). IGF-II transcription in skeletal myogenesis is controlled by mTOR and nutrients. J. Cell Biol..

[47] Ghaleb A.M., Yang V.W. (2017). Krüppel-like factor 4 (KLF4): What we currently know. Gene.

[48] Lee Y.I., Kim S.-J., Kim Y.I.L.-J. (1996). Transcriptional Repression of Human Insulin-Like Growth Factor-II P4 Promoter by Wilms’ Tumor Suppressor WT1. DNA Cell Biol..

[49] Sun D., Chen S., Cheng A., Wang M. (2016). Roles of the Picornaviral 3C Proteinase in the Viral Life Cycle and Host Cells. Viruses.

[50] Dimova D.K., Dyson N.J. (2005). The E2F transcriptional network: Old acquaintances with new faces. Oncogene.

[51] Babon J.J., Varghese L.N., Nicola N.A. (2014). Inhibition of IL-6 family cytokines by SOCS3. Semin. Immunol..

[52] Zhu M., Duan H., Gao M., Zhang H., Peng Y. (2015). Both ERK1 and ERK2 Are Required for Enterovirus 71 (EV71) Efficient Replication. Viruses.

[53] Luo H., Yanagawa B., Zhang J., Luo Z., Zhang M., Esfandiarei M., Carthy C., Wilson J.E., Yang D., McManus B.M. (2002). Coxsackievirus B3 Replication Is Reduced by Inhibition of the Extracellular Signal-Regulated Kinase (ERK) Signaling Pathway. J. Virol..

[54] Si X., Luo H., Morgan A., Zhang J., Wong J., Yuan J., Esfandiarei M., Gao G., Cheung C., McManus B.M. (2005). Stress-Activated Protein Kinases Are Involved in Coxsackievirus B3 Viral Progeny Release. J. Virol..

[55] Aguech-Oueslati L., Jaidane H., Sane F., Jrad-Battikh N., Ben Hamed S., Hober D., Gharbi J. (2017). Evaluation of Contamination Risks with Coxsackievirus B4 E2 in Swiss Albino Mice Stools. Curr. Microbiol..

[56] Kecha O., Martens H., Franchimont N., Achour I., Hazee-Hagelstein M.-T., Charlet-Renard C., Geenen V., Winkler R. (1999). Characterization of the Insulin-Like Growth Factor Axis in the Human Thymus. J. Neuroendocr..

[57] Kecha O. (2000). Involvement of Insulin-Like Growth Factors in Early T Cell Development: A Study Using Fetal Thymic Organ Cultures. Endocrinology.

[58] Steinmetz A.B., Johnson S.A., Iannitelli D.E., Pollonini G., Alberini C.M. (2016). Insulin-like growth factor 2 rescues aging-related memory loss in rats. Neurobiol. Aging.

[59] Qiu Q., Basak A., Mbikay M., Tsang B.K., Gruslin A. (2005). Role of pro-IGF-II processing by proprotein convertase 4 in human placental development. Proc. Natl. Acad. Sci. USA.

[60] Van Koetsveld P.M., Vitale G., Feelders R.A., Waaijers M., Sprij-Mooij D.M., De Krijger R.R., Hofland L.J., de Herder W.W., Lamberts S.W.J., de Krijger R.R. (2013). Interferon-β is a potent inhibitor of cell growth and cortisol production in vitro and sensitizes human adrenocortical carcinoma cells to mitotane. Endocr. Relat. Cancer.

[61] Booy S., van Eijck C.H.J., Janssen J.A.M.J.L., Dogan F., van Koetsveld P.M., Hofland L.J. (2015). IFN-β is a potent inhibitor of insulin and insulin like growth factor stimulated proliferation and migration in human pancreatic cancer cells. Am. J. Cancer Res..

[62] Henke A., Mohr C., Sprenger H., Graebner C., Stelzner A., Nain M., Gemsa D. (1992). Coxsackievirus B3-induced production of tumor necrosis factor-alpha, IL-1 beta, and IL-6 in human monocytes. J. Immunol..

[63] Yang K., Puel A., Zhang S., Eidenschenk C., Ku C.-L., Casrouge A., Picard C., Von Bernuth H., Senechal B., Plancoulaine S. (2005). Human TLR-7-, -8-, and -9-Mediated Induction of IFN-α/β and -λ Is IRAK-4 Dependent and Redundant for Protective Immunity to Viruses. Immunity.

[64] Hober D., Chehadeh W., Bouzidi A., Wattré P. (2001). Antibody-Dependent Enhancement of Coxsackievirus B4 Infectivity of Human Peripheral Blood Mononuclear Cells Results in Increased Interferon-α Synthesis. J. Infect. Dis..

[65] Jaïdane H., Gharbi J., Lobert P.-E., Caloone D., Lucas B., Sane F., Idziorek T., Romond M.-B., Aouni M., Hober D. (2008). Infection of primary cultures of murine splenic and thymic cells with coxsackievirus B4. Microbiol. Immunol..

