# Supplementary material for: Modulation of IGF2 Expression in the Murine Thymus and Thymic Epithelial Cells Following Coxsackievirus-B4 Infection
Source: Microorganisms. 2021 Feb 15;9(2):402. doi: 10.3390/microorganisms9020402 (PMC7919294; doi:10.3390/microorganisms9020402)
Supplement: Supplementary file 1 [file microorganisms-09-00402-s001.pdf]

microorganisms

Supplementary data

Modulation of IGF2 expression in the murine thymus and Thymic Epithelial Cells Following Coxsackievirus-B4 Infection

Hélène Michaux[1], Aymen Halouani[2], Charlotte Trussart[1], Chantal Renard[1], Hela Jaïdane[2], Henri Martens[1], Vincent Geenen[1]*†, Didier Hober[3]*†

[1] GIGA-I3 Center of Immunoendocrinology, GIGA Research Institute, University of Liège, 4000 Liège, Belgium; hmichaux88@gmail.com (H.M.); charlotte.trussart@uliege.be (C.T.); ach.charlet@outlook.be (C.R.); hmartens@uliege.be (H.M.)
[2] Laboratoire des Maladies Transmissibles et Substances Biologiquement Actives LR99ES27, Université El Manar (Tunis) and Université de Monastir, 5000 Monastir, Tunisia; halouani.aymen@yahoo.com (A.H.); jaidanehela@yahoo.fr (H.J.)
[3] Laboratoire de Virologie ULR3610, Université de Lille CHU Lille, F-59000 Lille, France
* Correspondence: didier.hober@chru-lille.fr (D.H.) ; vgeenen@uliege.be (V.G.).
† These senior coauthors contributed equally to the manuscript.

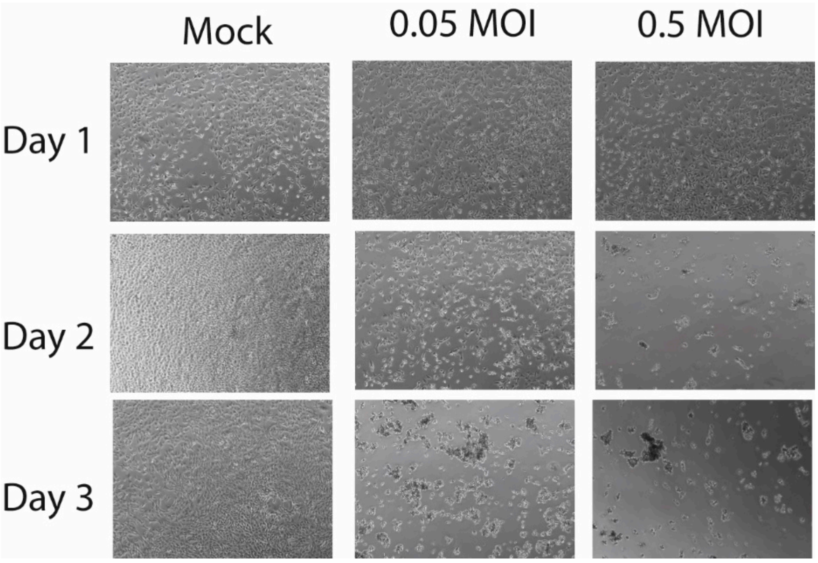

Figure 1. Microscopic examination of mock- and Coxsackievirus B4 (CV-B4)-infected MTE4-14 cells 1–3 days postinfection (P.I.).

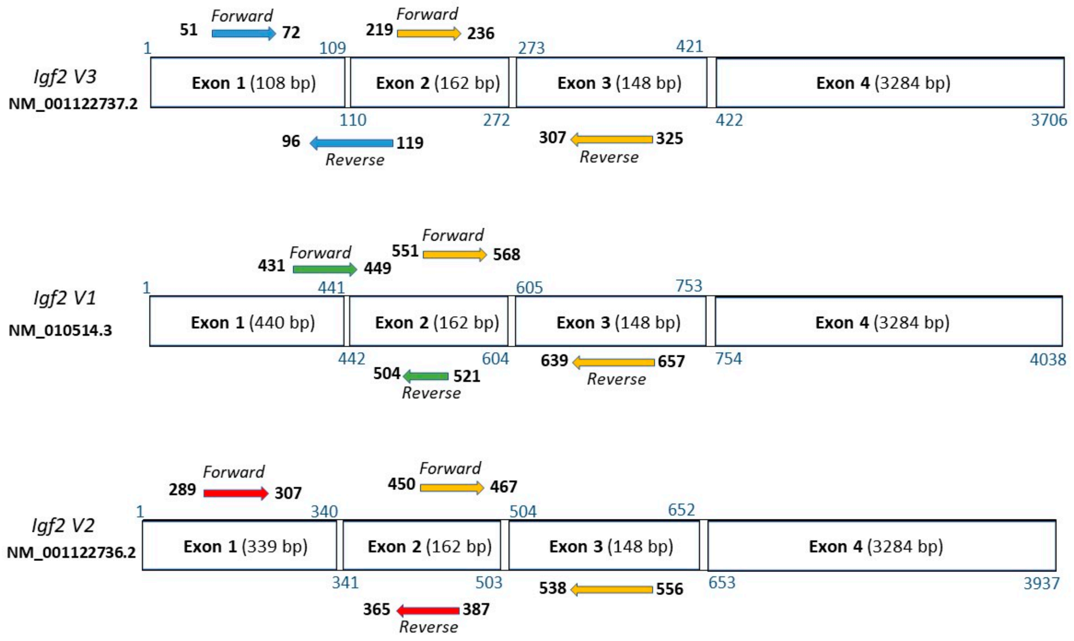

Figure 2. A. Primer localization in insulin-like growth factor 2 (Igf2) mRNA transcripts. Blue arrows indicate forward and reverse primer localization for *Igf2* V3, green arrows indicate forward and reverse primer localization for *Igf2* V1, red arrows indicate forward and reverse primer localization for *Igf2* V2 and orange arrows indicate forward and reverse primer localization for *total Igf2* detection on each transcript. *Igf2* V1, V2 and V3 exon sizes are indicated in base pair (bp). Blue numbers indicate nucleotide number. NCBI transcript accession number is indicated in bold type under the name of each transcript.

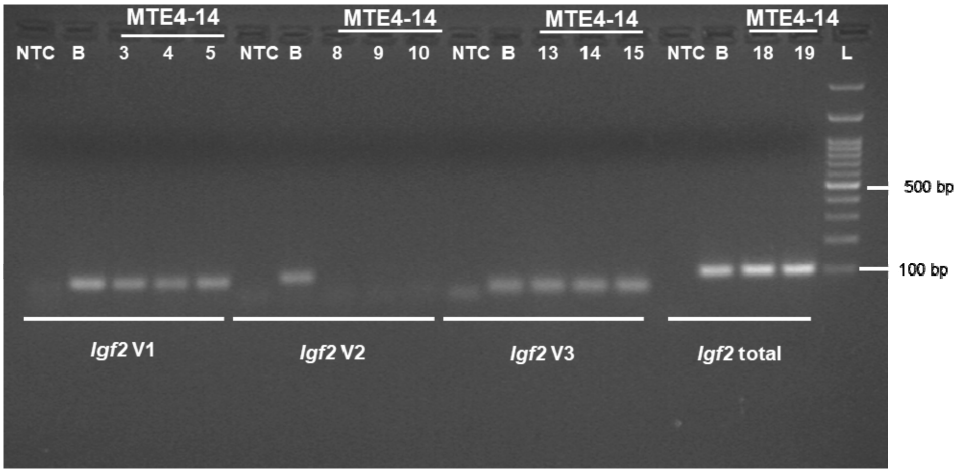

Figure 2. B. Uncropped whole PCR gel for Figure 1A. NTC, no template control. B, brain positive control. Lanes 3–5, 8–10, and 18–19, MTE4-14 cells. L, O'GeneRuler 100 bp DNA ladder. **Weak band in no** template control (50 bp) is primer dimers amplification.

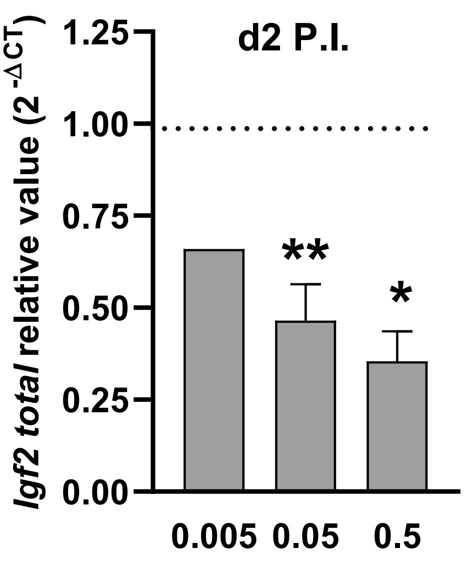

Figure 3. A. Relative mRNA expression of the *total Igf2* mRNA in CV-B4-infected cells (multiplicity of infection (MOI) = 0.005 to MOI = 0.5) 2 days P.I. **Data are represented as mean of $2^{-\Delta CT}$ ± SEM.** Mock samples are represented as a dashed line set at y = 1. **MOI = 0.005, n = 1; MOI = 0.05, n = 12; MOI = 0.5, n = 4. Ratio paired *t*-test, ****p* < 0.05 and *****p*** < 0.01.

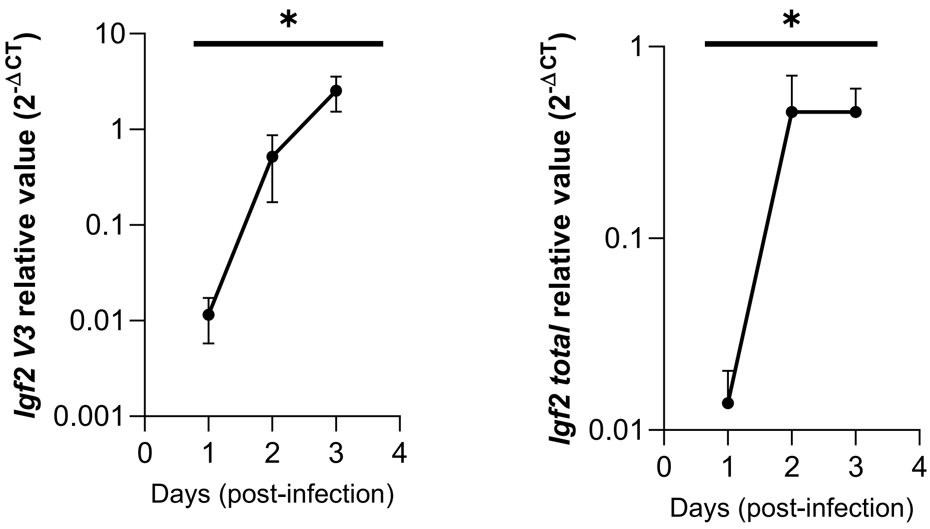

Figure 3. B. Relative mRNA expression of *Igf2 V3* and total *Igf2* mRNA in mock-infected MTE4-14 cells 1, 2, and 3 days P.I. **Data are represented as mean of $2^{-\Delta CT}$ ± SEM; n = 5–10, Welch's ANOVA test. **p* < 0.05.**

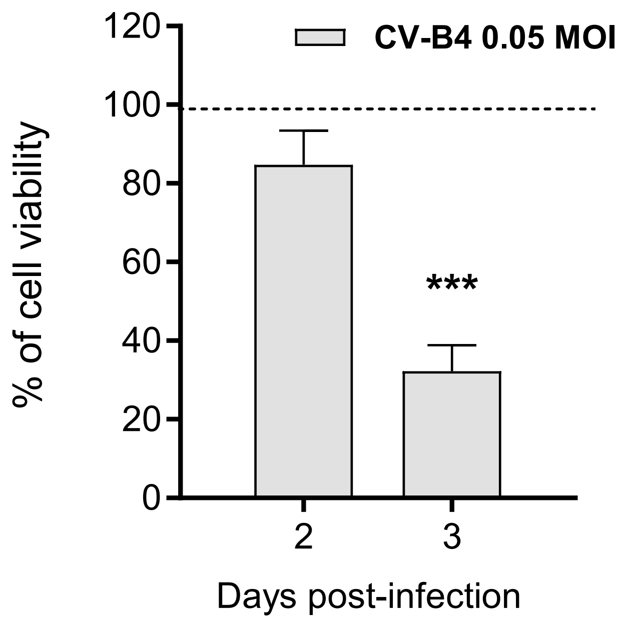

Figure 3. C. 3-(4,5-Dimethyl-2-thiazolyl)-2,5-diphenyl-2H-tetrazolium bromide reagent (MTT) cell viability assay analysis on day 2 and 3 P.I. CV-B4-infected cells relative to matched mock-infected cells (represented as a dashed line set at y = 100) are shown. Data are represented as the mean of fold change ± SEM; n = 7–11, ratio paired *t* test, ***$p < 0.001$.

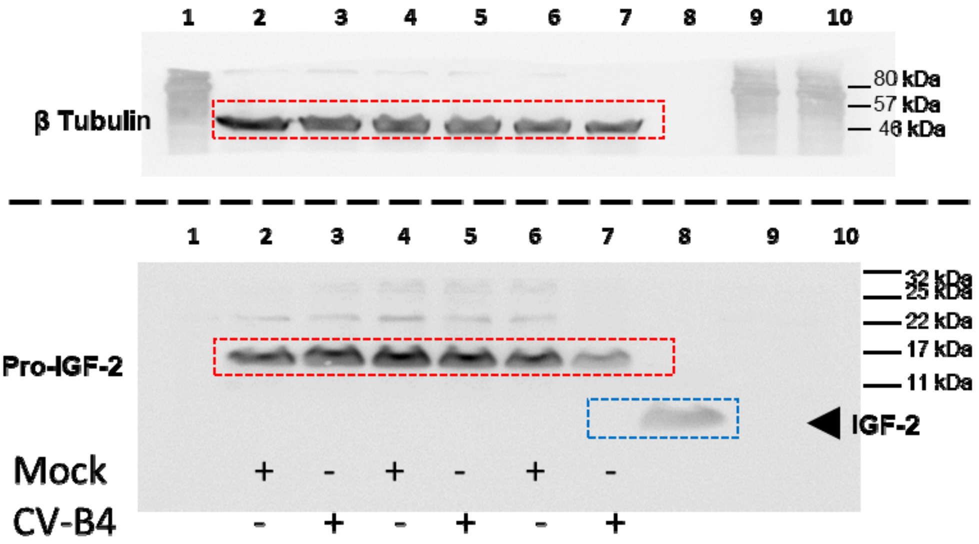

Figure S3D. Full-length Western blot for Figure 2C. Membranes were cut horizontally into two parts prior to immunostaining for β-tubulin and IGF-2. Lanes 1, 9, and 10, Color-coded Prestained Protein Marker, Broad Range (11–250 kDa). Lanes 2, 4, 6: mock-infected cells; Lanes 3, 5, and 7: CV-B4 infected cells. Lane 8, IGF-2 positive control.

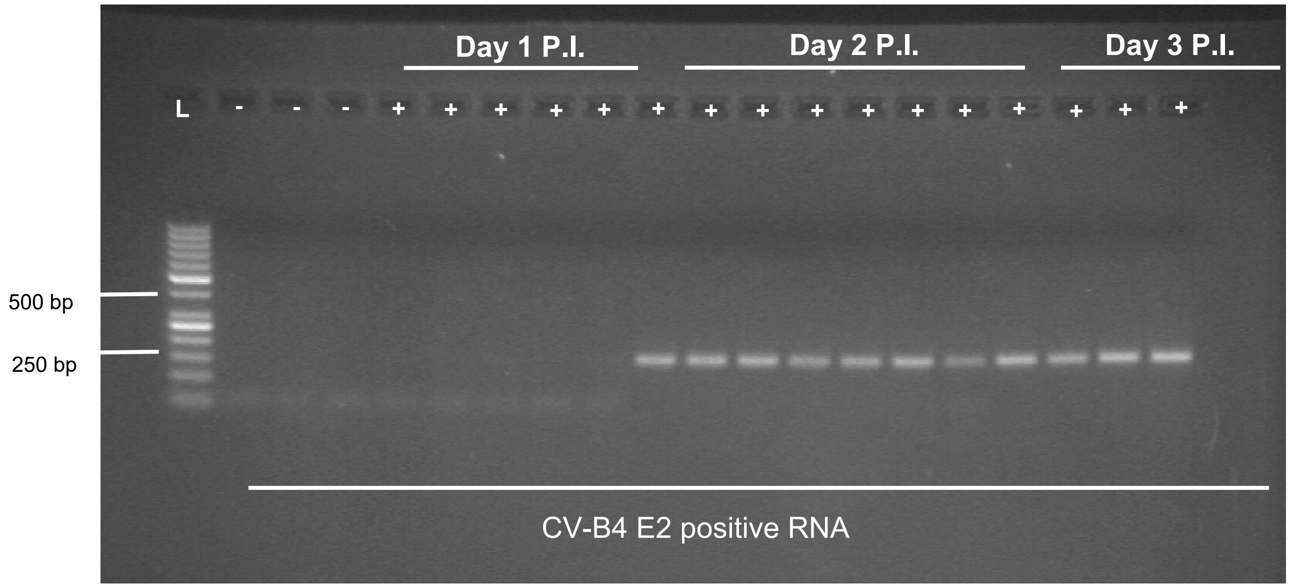

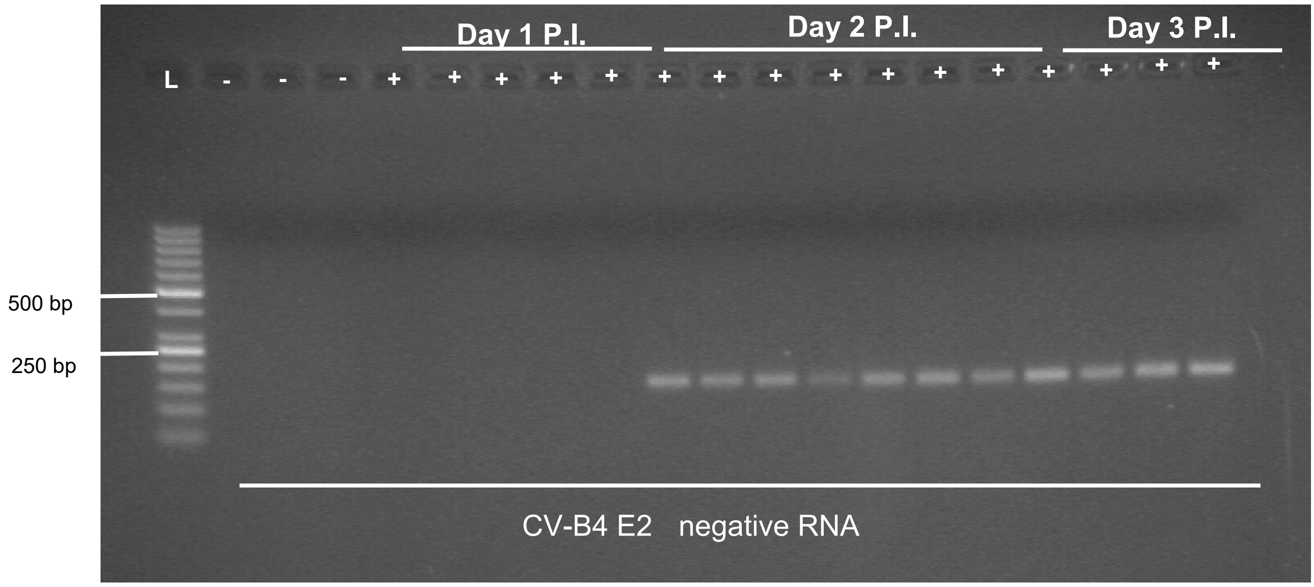

Figure S3E. Uncropped whole PCR gel for Figure 2D. L, O'GeneRuler 50 bp DNA ladder; (-): Mock-infected cells and (+): CV-B4-infected cells.

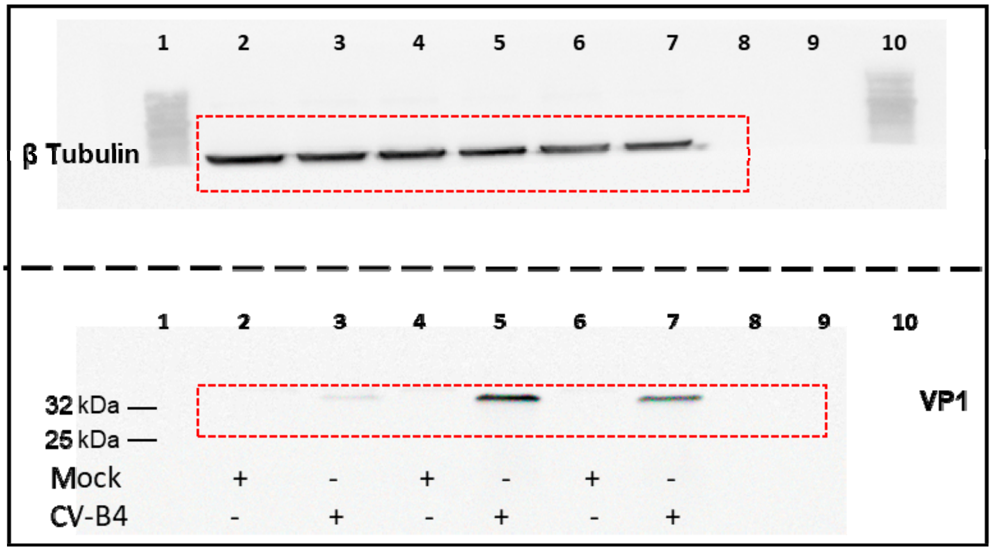

Figure 3. F. Full-length Western blot for Figure 2E. Membranes were cut horizontally into two parts prior to immunostaining for β Tubulin and VP1. Lanes 1 and 10, Color-coded Prestained Protein Marker, Broad Range (11–250 kDa). Lanes 2, 4, and 6: mock-infected cells; Lanes 3, 5, and 7: CV-B4 infected cells. Lanes 8 and 9, RIPA buffer only.

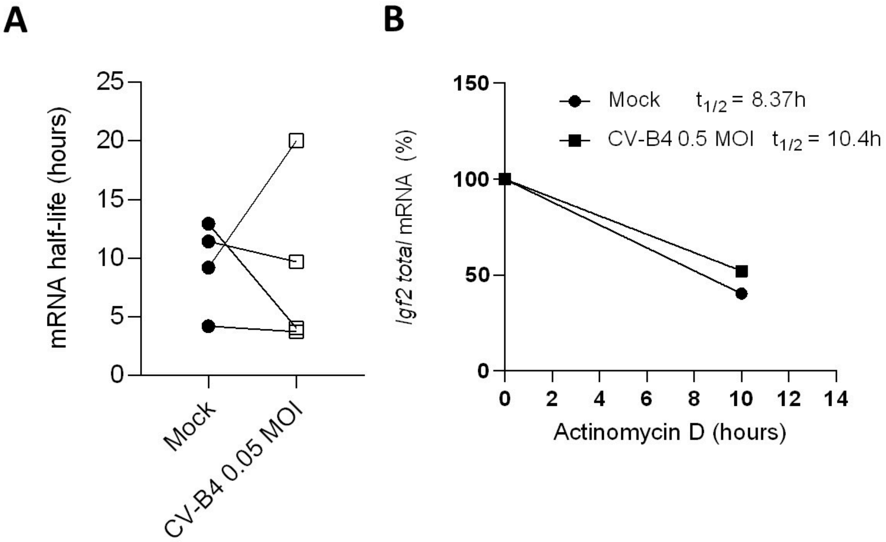

Figure 4. Effect of CV-B4 on *Igf2* transcript stability. (A) mRNA half-life of *Igf2* V3 transcripts in CV-B4 (MOI = 0.05) or mock-infected cells at day 2 P.I. followed by 2-10 hours of treatment with actinomycin D; lines connect both mock and CV-B4 mRNA half-life values obtained from the same experiment, ascending lines indicate an increase of mRNA half-life values and descending lines indicate a decrease of mRNA half-life values; n = 4. (B) Relative mRNA expression of *total Igf2* mRNA in mock- or in CV-B4-infected cells (MOI = 0.05) on day 2 P.I. followed by 10 hours of treatment with actinomycin D; n = 1. (A, B) A vehicle control was used for data normalization; (B) RT-qPCR was performed in parallel with standard plasmids with a known copy number for *total Igf2* and *Hprt*. The copy number of *Igf2* was normalized to that of *Hprt* for each sample. (A) Ratio paired t-test, $p > 0.05$.

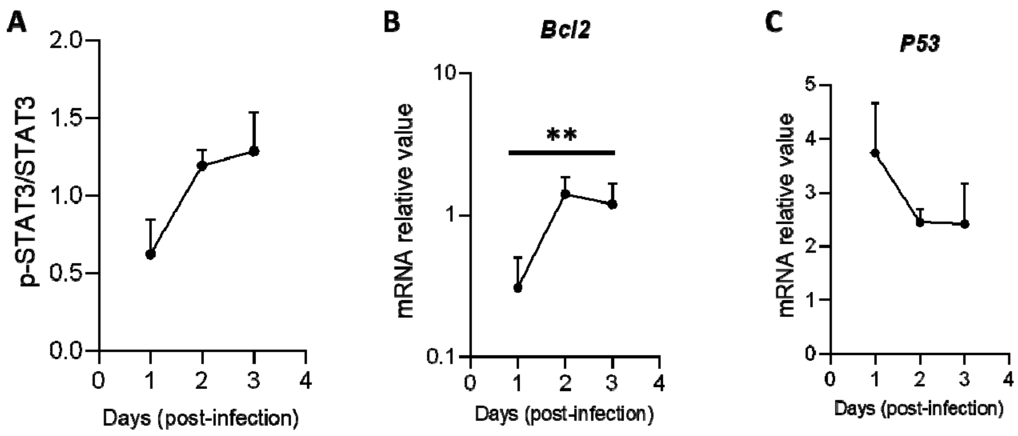

Figure 5. Parallel increase in signal transducer and activator of transcription 3 (STAT3)^{pY705} with *Bcl2* in mock cells. (A) STAT3^{pY705} relative quantification in mock-infected cells 1, 2, and 3 days P.I. Data are represented as mean ± **SEM**, n = 4–5. (B) Relative mRNA expression of *Bcl2* and (C) *P53* mRNA in mock-infected MTE4-14 cells 1, 2, and 3 days P.I. **Data are represented as mean of $2^{-\Delta CT}$ ± SEM;** A-C, **n = 3–6;** one-way ANOVA, **$^{**}p < 0.01$.**

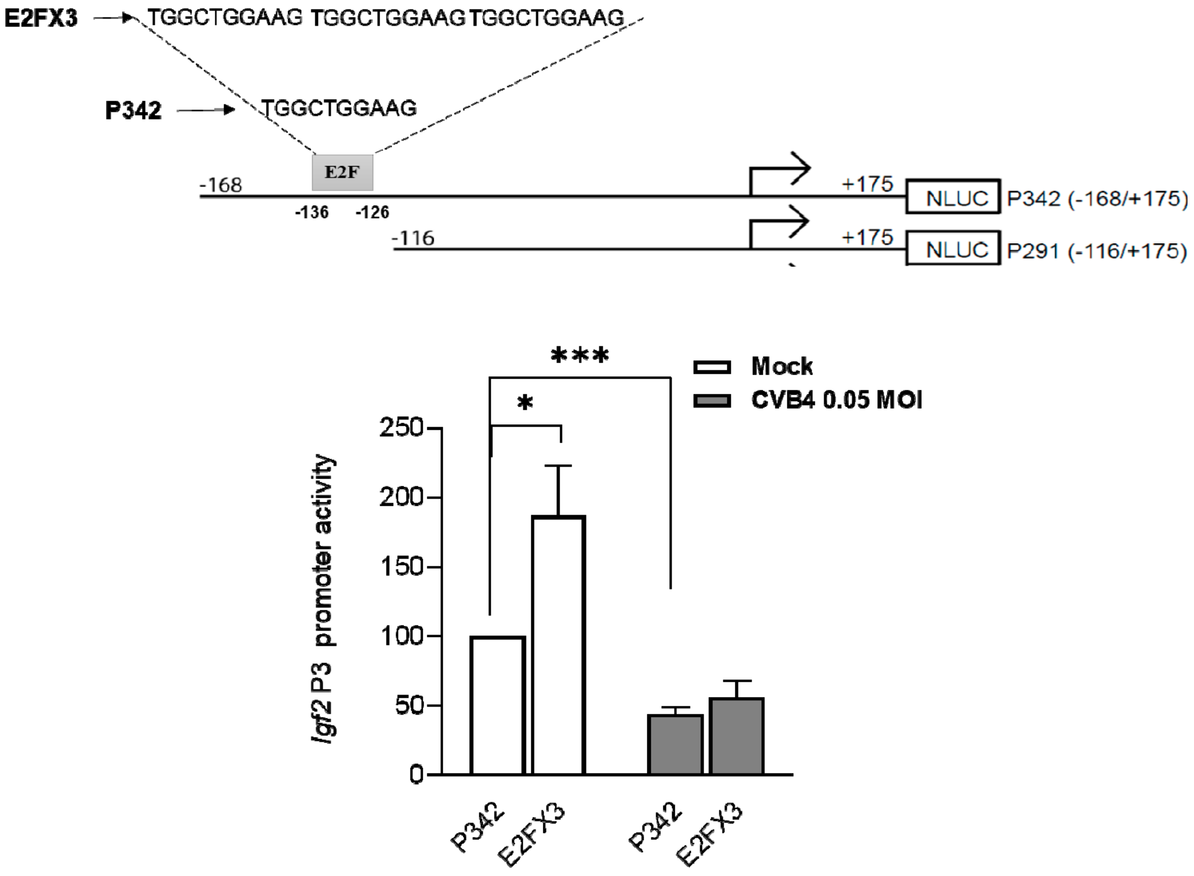

Figure 6. Effect of CV-B4 on *Igf2* P3 promoter with the E2FX3 construct. Top, schematic structure of *Igf2* P3 promoter with three sequential E2F binding sites (E2FX3) and wild type *Igf2* P3 (P342) with one E2F binding site. Bottom, Nanoluciferase relative activity of *Igf2* P3 promoter constructs on day 2 P.I. in CV-B4- (MOI = 0.05) or mock-infected cells transfected with the full *Igf2* P3 promoter construct (P342) or with *Igf2* P3 promoter containing three binding sites for E2F (E2FX3). The mean of the relative dual-luciferase activity normalized to mock is represented as ± SEM. Mock-infected cells

transfected with the full *Igf2* P3 promoter (P342) is set at 100% in each experiment; n = 5–6. Ratio paired *t*-test, *$p < 0.05$ and ***$p < 0.001$.

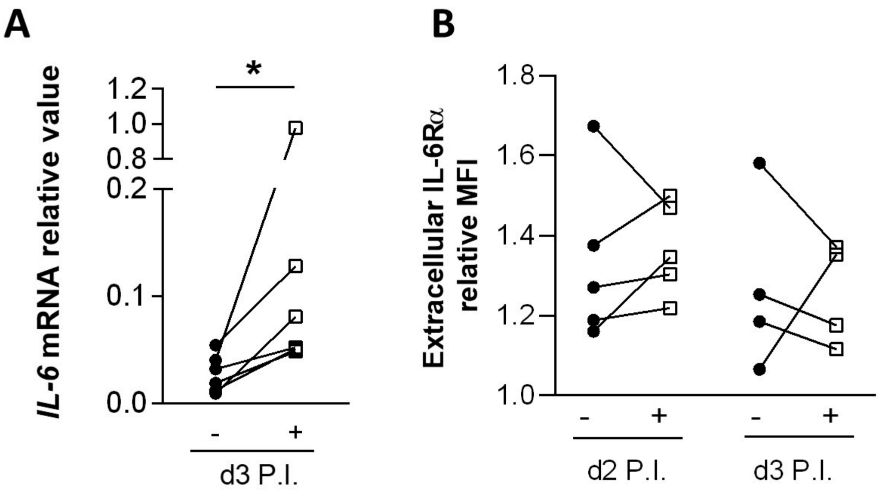

Figure 7. *Il6* mRNA relative expression and flow cytometry analysis of extracellular IL-6Rα in mock- (-) or in CV-B4-infected cells (+). (A) n = 6; **Wilcoxon test, *p < 0.05**. (B) Each relative Mean of Fluorescence Intensity (MFI) value represents MFI value of the sample divided by the corresponding isotype control MFI value; n = 4-5. **Ratio paired *t*-test,** $p > 0.05$**.**

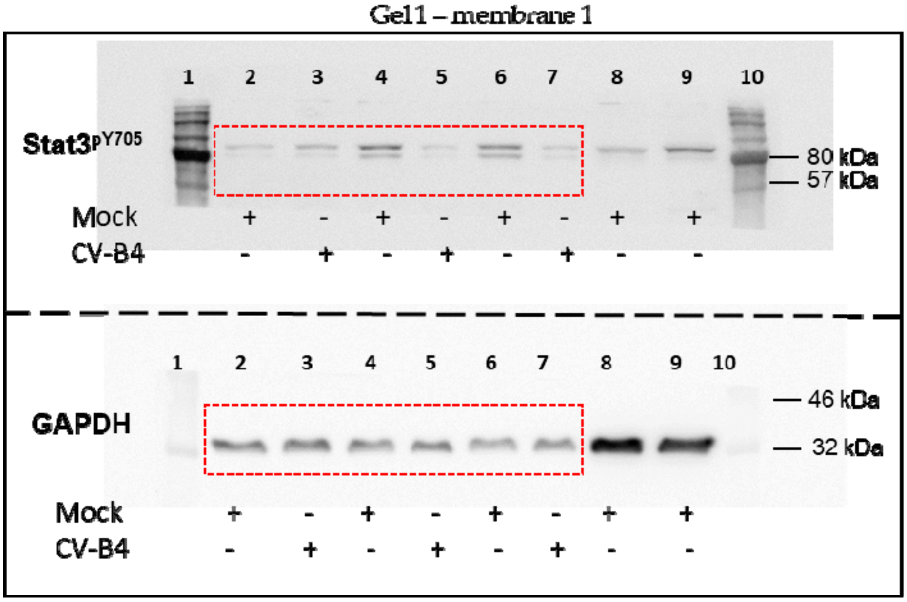

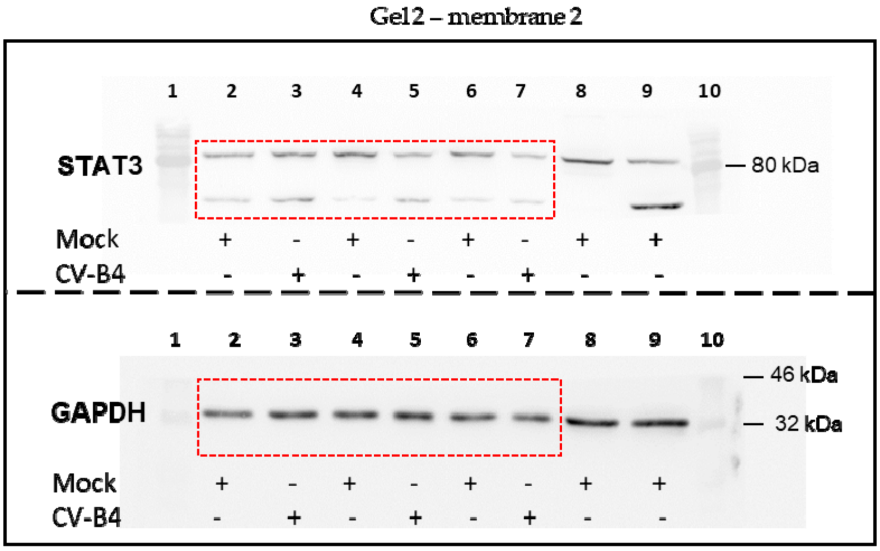

Figure 8. A. Full-length Western blot for Figure 5A. Membranes were cut horizontally into two parts prior to immunostaining of GAPDH and STAT3 (or STAT3 ^{pY705} and GAPDH). Lanes 1 and 10, Color-coded Prestained Protein Marker, Broad Range (11–250 kDa). Lanes 2, 4, and 6: mock-infected cells; Lanes 3, 5, and 7: CV-B4 infected cells. Lane 8: mock-infected MTE4-14 cells; lane 9: mock-infected MTE4-14 cells stimulated with IL-6 for 30 min.

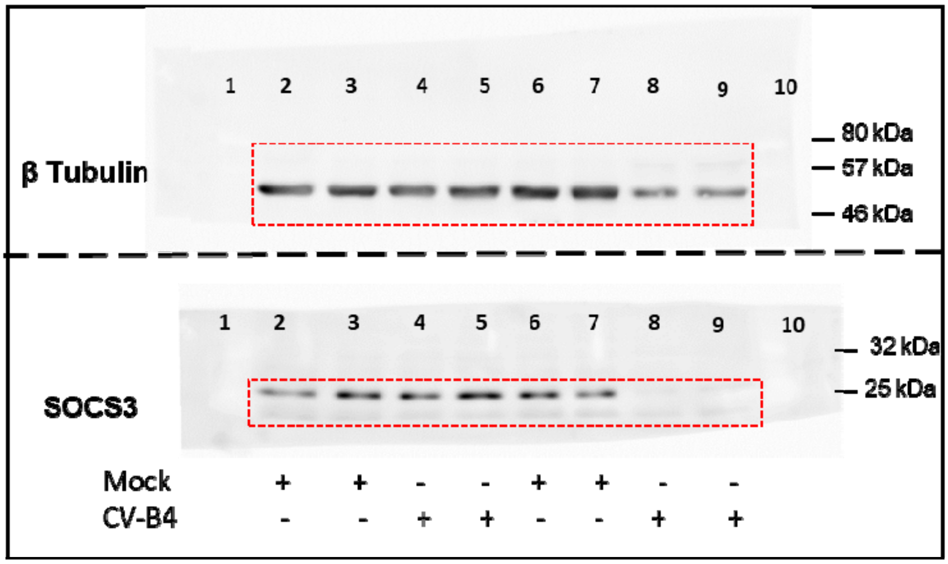

Figure 8. B. Full-length Western blot for Figure 5C. Membranes were cut horizontally into two parts prior to immunostaining for βTubulin and SOCS3. Lanes 1 and 10, Color-coded Prestained Protein Marker, Broad Range (11–250 kDa). Lanes 2, 3, 6, 7: mock-infected cells; lanes 4, 5, 8, 9: CV-B4-infected cells.

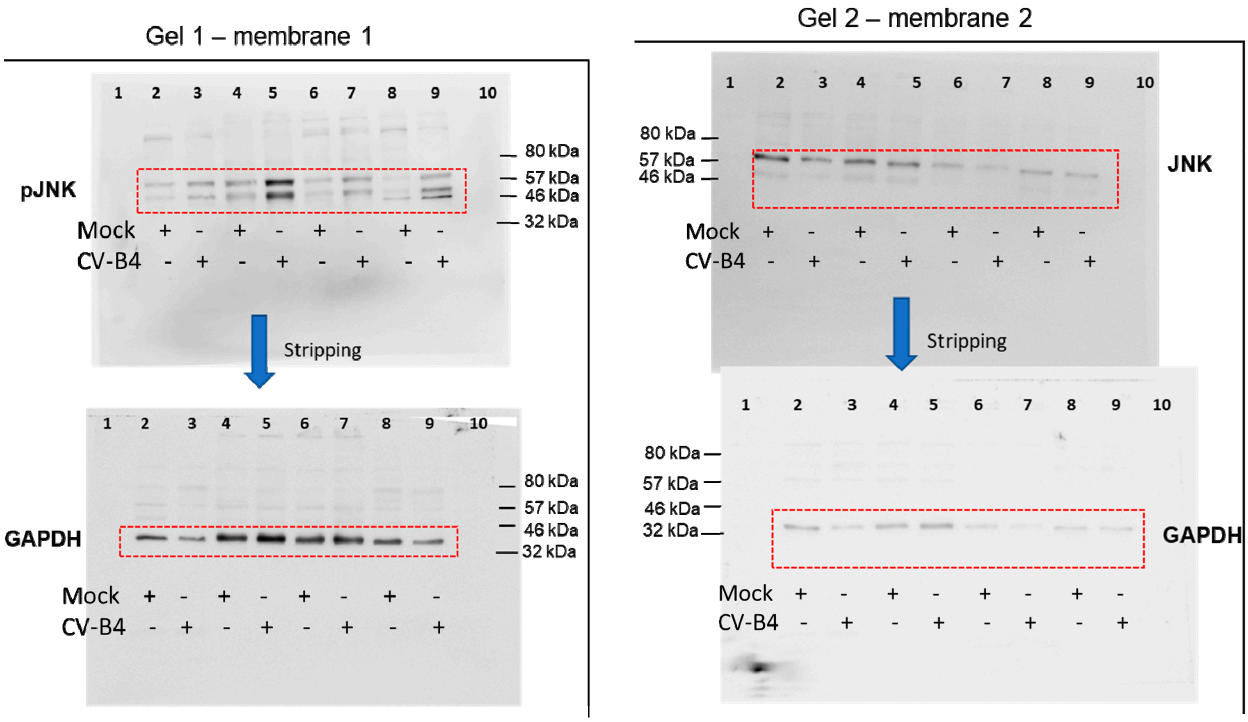

Figure 8. C. Full-length **W**estern blot for Figure 5D. pJNK or JNK staining is performed followed by membrane stripping prior to GAPDH immunostaining. Lanes 1 and 10, Color-coded Prestained Protein Marker, Broad Range (11–250 kDa). Lanes 2, 4, 6, and 8: mock-infected cells; lanes 3, 5, 7, and 9: CV-B4-infected cells.

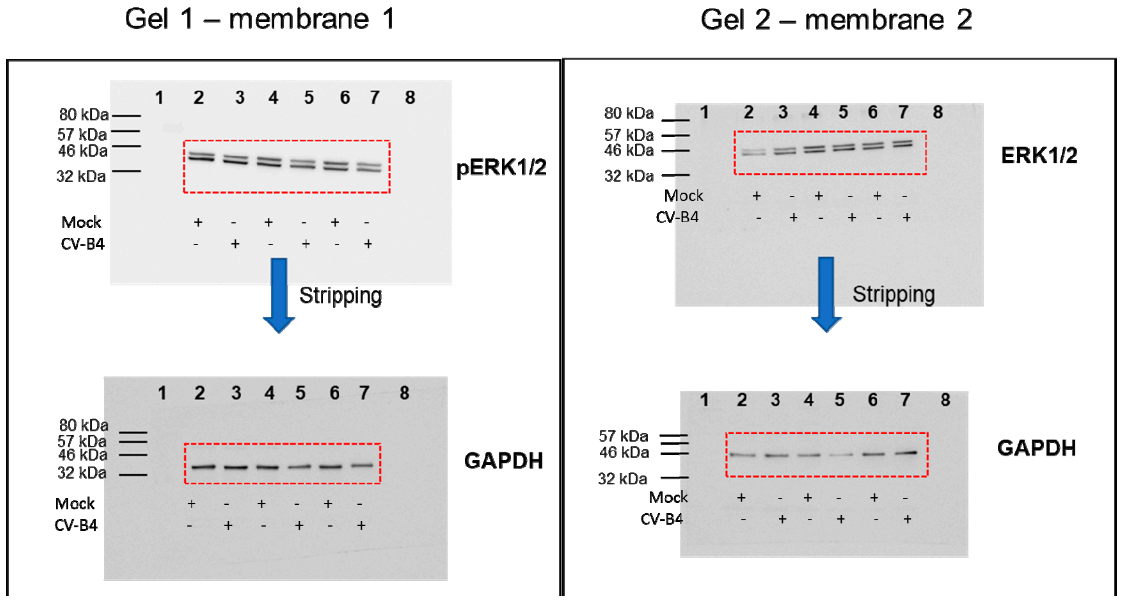

Figure 8. D. Full-length **W**estern blot for Figure 5E. pERK or ERK staining is performed followed by membrane stripping prior to GAPDH immunostaining. Lanes 1 and 10, Color-coded Prestained Protein Marker, Broad Range (11–250 kDa). Lanes 2, 4, and 6: mock-infected cells; lanes 3, 5, and 7: CV-B4-infected cells.

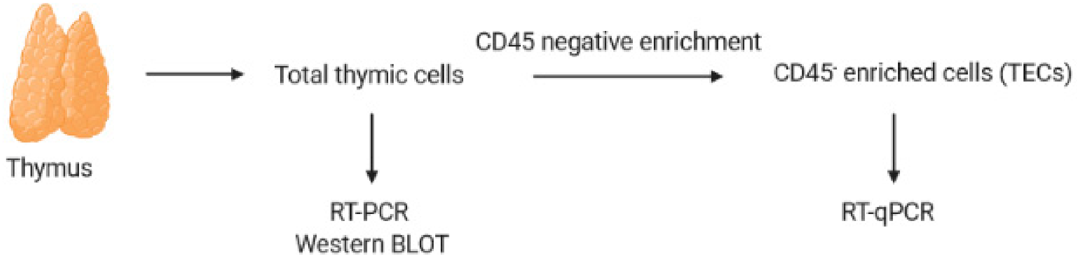

Figure 9. A. Thymus processing steps for isolation of total thymic cells (unsorted cells) and CD45-negative enriched cells (TECs).

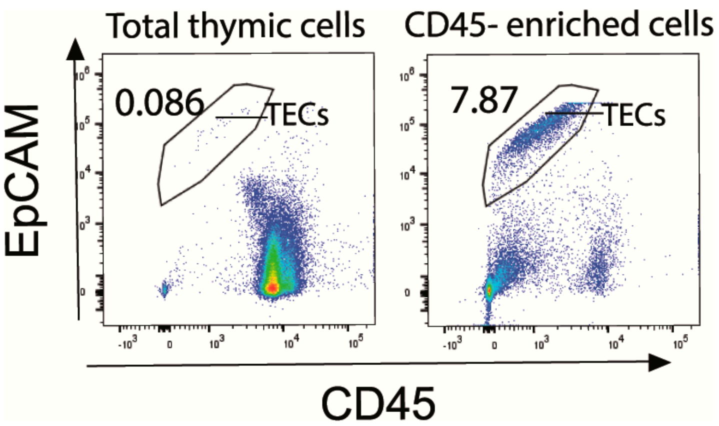

Figure 9. B. **Flow cytometry analysis of CD45-negative enriched TECs (EpCAM**+CD45−) *versus* the unsorted total thymic population. The numbers indicate the percentage of TECs population.

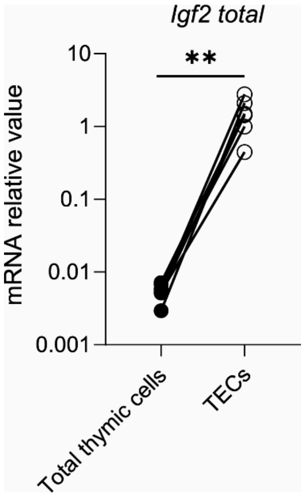

Figure 9. C. Relative mRNA expression of the *total Igf2* mRNA in the unsorted total thymic population and matched enriched TECs fraction (CD45−). Mock-inoculated mice (n = 6) on day 2 P.I. were used. Student's *t*-test, ***p* < 0.01.

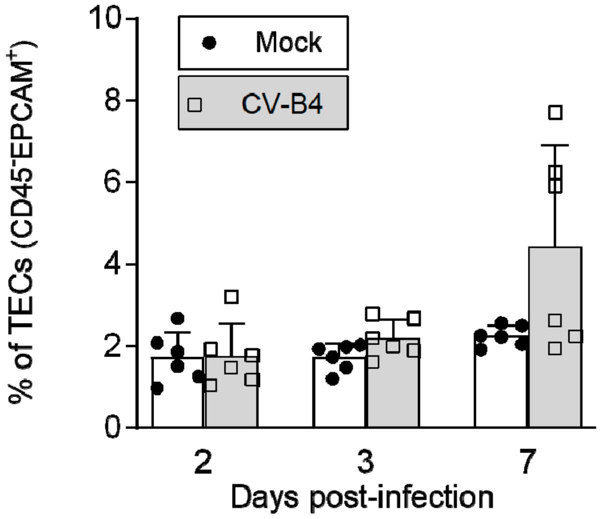

Figure 9. D. Flow cytometry analysis of total thymic cells 2, 3, and 7 days P.I. Gating strategy was performed as indicated in Figure S9B. Histogram represents the mean of relative value ± SD; n = 6. Kruskal–Wallis test, $p > 0,05$.

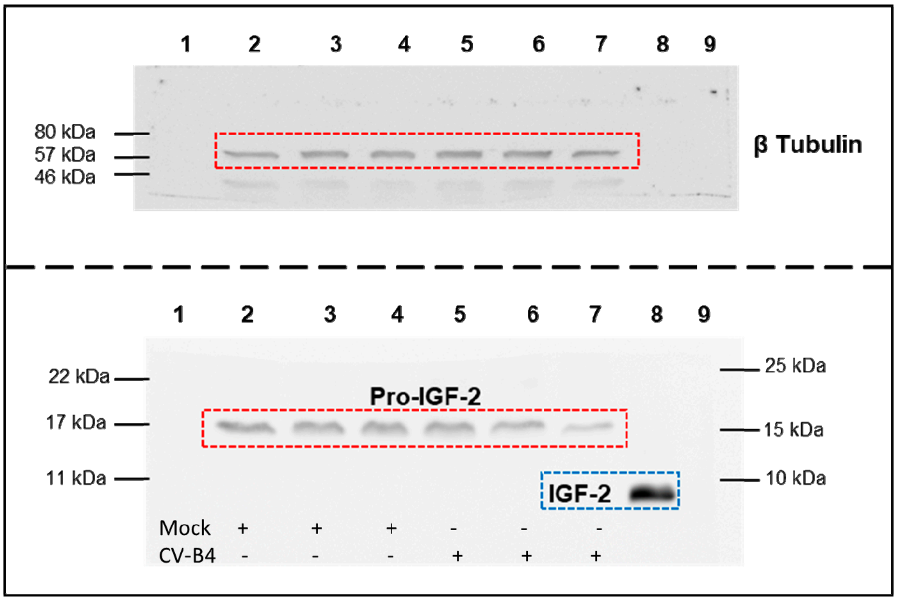

Figure 9. E. Full-length Western blot for Figure 6C. Membranes were cut horizontally into two parts prior to immunostaining for β Tubulin and IGF-2. Lane 1, Color-coded Prestained Protein Marker, Broad Range (11–250 kDa) and lane 9, Spectra™ Multicolor Low Range Protein Ladder). Lanes 2, 3, and 4: mock-inoculated mice; lanes 5, 6, and 7: CV-B4-inoculated mice. Lane 8, IGF-2 positive control.

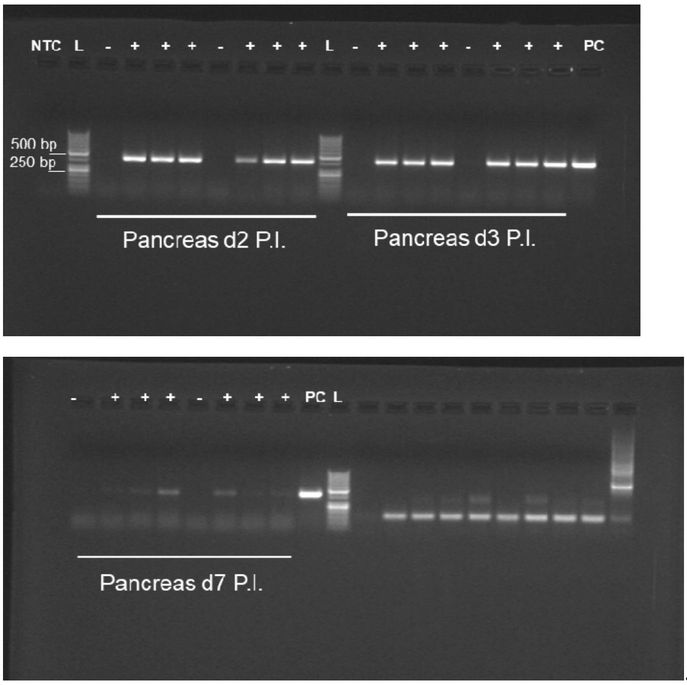

Figure 9. F. Uncropped whole PCR gels for Figure 6D. NTC, no template control; L, O'GeneRuler 50 bp DNA ladder; PC, positive control (MTE4-14 cells infected with CV-B4). (-): mock-inoculated mice and (+): CV-B4- inoculated mice. Undescribed wells are from an unrelated experiment.

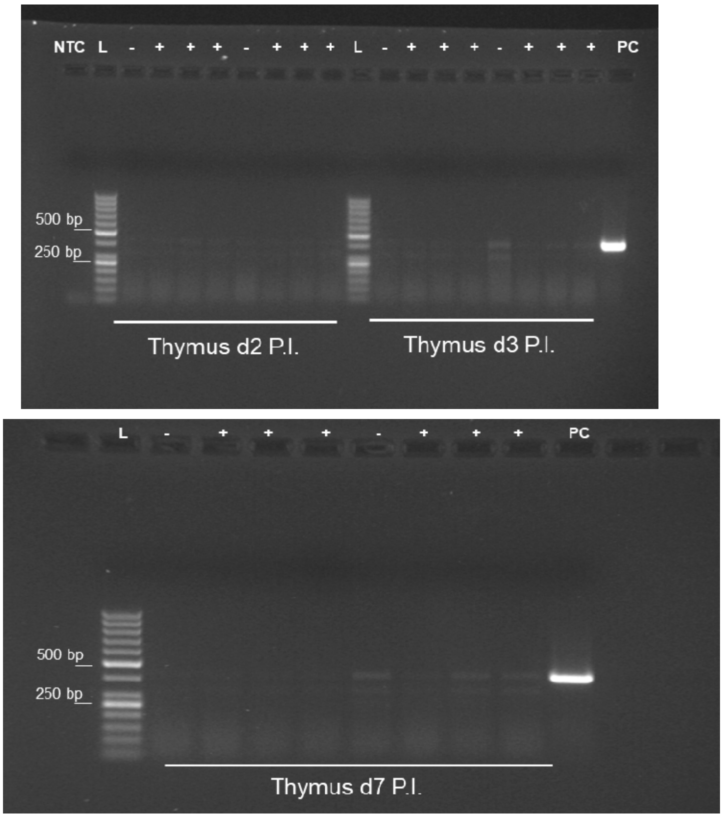

Figure 9. F. Uncropped whole PCR gels for Figure 6D. NTC, no template control; L, O'GeneRuler 50 bp DNA ladder; PC, positive control (MTE4-14 cells infected with CV-B4). (-): mock-inoculated mice and (+): CV-B4-inoculated mice. Undescribed wells are from an unrelated experiment.

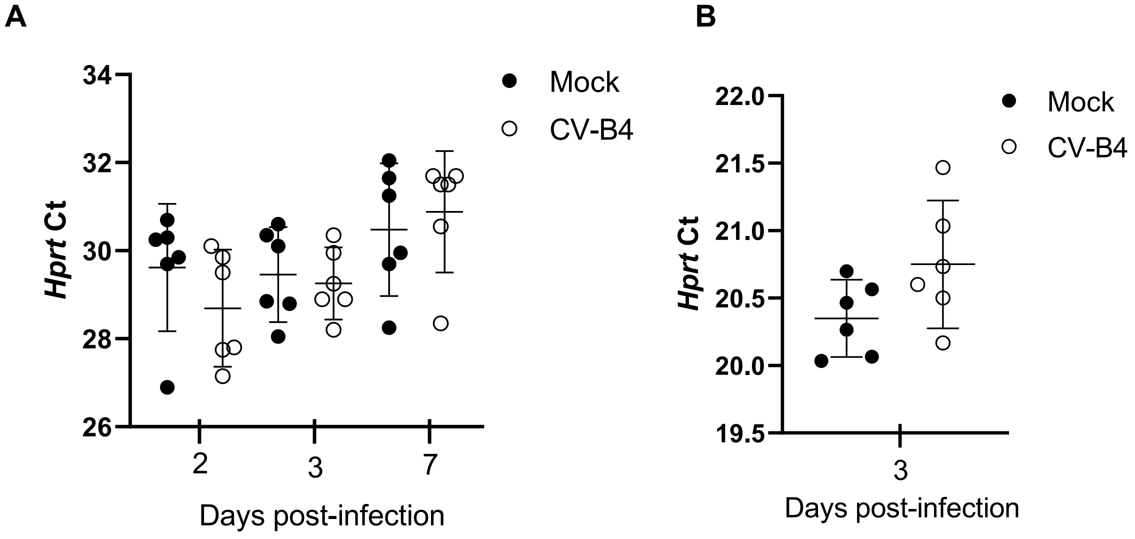

Figure 10. ***Hprt*** *Cycle threshold* in mice (A) and in MTE4-14 cells (B). (A), unpaired two-tailed t-test p>0.05 and (B) paired two-tailed t-test p>0.05.

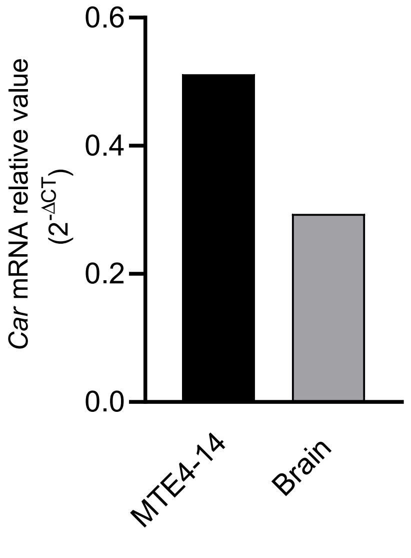

Figure 11. Coxsackievirus and adenovirus receptor (CAR) mRNA expression in MTE4-14 cells . The mean **of $2^{-\Delta CT}$ is shown,** n = 1. Murine brain is used as the positive control. Gene expression is calculated based on the comparative Ct normalized to that of *β-actin.*

Day 2 P.I.	Ct *Hprt*	Ct *total Igf2*	Ct *Igf2* V3	Ct *Igf2* V1	Ct *Igf2* V2
**Mock n°1**	N/A	29,7	31,0	31,4	34,4
	29,7	29,8	31,3	31,0	34,5
		29,6	30,7	32,3	
**Mock n°2**	30	29,2	30,7	31,1	32,8
	29,7	29,4	30,4	31,8	34,4

		29,2	30,5	31,3	
	27	28,0	31,0	29,6	32,7
**Mock n°3**	26,8	28,0	30,8	29,7	31,8
		28,2	30,3	29,6	
**Mock n°4**	30	29,6	31,0	31,0	33
	31,4	29,6	30,4	31,0	32,5
		29,7	31,6	30,8	
**Mock n°5**	29,8	29,5	31,1	31,2	32,6
	30,7	29,8	31,6	31,3	32,6
		29,9	31,2	31,6	
	29,9	28,9	30,8	30,1	33,6
**Mock n°6**	30,7	28,9	30,7	29,8	35,2
		28,7	30,8	31,3	
**CV-B4 n°1**	26,6	28,5	30,1	29,8	34,4
	27,7	28,6	30,5	30,1	33
		28,6	30,6	30,6	
**CV-B4 n°2**	27,6	28,1	30,4	29,6	33,2
	28	28,7	30,1	30,0	32,7
		28,6	30,4	29,9	
**CV-B4 n°3**	27,7	27,8	30,0	29,8	32,7
	27,8	27,9	30,2	29,3	32,9
		27,8	30,3	29,5	
**CV-B4 n°4**	30,6	29,1	31,0	29,5	33,4
	29,1	28,2	31,0	30,3	33,6
		28,5	31,4	30,2	
**CV-B4 n°5**	30	29,3	31,1	30,2	37,3
	30,2	29,5	31,1	31,2	36,8
		29,8	31,8	31,0	
**CV-B4 n°6**	29,3	29,2	30,8	30,7	37,3
	29,7	29,3	31,3	31,5	N/A
		29,3	31,2	30,6	

Day 3 P.I.	Ct *Hprt*	Ct *total Igf2*	Ct *Igf2* V3	Ct *Igf2* V1	Ct *Igf2* V2
**Mock n°1**	27,8	28,8	31,6	30,7	33,6
	28,3	28,8	31,5	30,6	N/A
		28,5	31,2	30,5	
**Mock n°2**	28,6	28,6	30,4	31,7	34,3
	29,1	28,8	30,8	32,0	

		29,0	30,7	31,6	
	28,7	28,8	31,2	30,9	34,1
**Mock n°3**	28,9	28,6	30,9	31,0	
		28,5	31,4	30,8	
**Mock n°4**	30,6	29,5	31,5	31,9	33,1
	30,6		30,7	32,8	33,4
		29,5	31,3	31,6	
**Mock n°5**	30,1	30,6	25,8	32,9	35,7
	30,1	30,8	26,1	32,8	34,4
		30,6	26,0	33,7	
	30,4	29,4	30,7	31,3	33,7
**Mock n°6**	30,3	29,5	31,0	31,7	N/A
		29,5	31,7	32,2	
**CV-B4 n°1**	27,6	28,3	30,5	29,9	32,7
	28,8	28,6	30,8	29,9	35
		28,4	30,1	30,4	
**CV-B4 n°2**	28,8	27,0	30,3	29,0	31,8
	29	27,4	30,1	29,5	32,6
		27,4	30,5	29,2	
**CV-B4 n°3**	28,8	28,3	31,2	29,5	32,8
	29	28,3	30,9	29,3	32,8
		28,0	30,7	29,6	
	28,9	28,6	27,7	29,8	32,5
**CV-B4 n°4**	29,6	28,9	27,9	30,4	32,6
		28,8	28,0	30,7	
**CV-B4 n°5**	30,1	29,6	30,4	30,3	34,2
	30,6	29,4	30,4	30,7	N/A
		29,8	31,0	31,2	
**CV-B4 n°6**	30	27,2	29,2	28,7	31,1
	29,9	27,0	29,6	29,3	31,8
		27,1	29,6	29,2	

**Day 7 P.I.**	**Ct *Hprt***	**Ct *total Igf2***	**Ct *Igf2* V3**	**Ct *Igf2* V1**	**Ct *Igf2* V2**
**Mock n°1**	30	29,4	28,7	31,7	35,8
	29,9	29,6	28,6	32,1	33,8
		29,7	29,0	32,1	
**Mock n°2**	28	27,5	27,2	29,8	30,6

Sum of	*Total Igf2*	*Igf2 V3*	*Igf2 V1*	*Igf2V2*	
	28,5	27,7	27,1	29,6	32,1
		27,6	27,3	30,2	
	31,2	30,3	30,3	33,2	34,5
**Mock n°3**	31,3	29,9	30,6	32,6	34,6
		29,7	30,6	32,7	
**Mock n°4**	29,9	29,2	30,8	30,7	33,1
	29,5	29,5	31,8	30,8	32,5
		29,4	31,4	31,3	
**Mock n°5**	31,6	29,8	31,4	31,5	n/a
	31,7	29,8	30,3	31,9	n/a
		30,3	30,7	32,1	
	32,1	30,6	30,6	33,2	n/a
**Mock n°6**	32	30,7	30,6	33,0	n/a
		31,2	30,6	32,6	
**CV-B4 n°1**	28,3	27,8	25,9	32,5	34,3
	28,4	28,1	26,0	32,6	32,4
		28,2	26,0	33,6	
**CV-B4 n°2**	32,4	30,2	27,9	33,8	n/a
	30,6	30,5	28,0	33,1	36,3
		30,7	27,8	33,7	
**CV-B4 n°3**	30,3	29,0	31,0	31,8	32,9
	30,8	29,4	31,6	32,5	n/a
		29,1	30,9	31,6	
**CV-B4 n°4**	31,2	30,6	30,7	30,2	n/a
	31,8	31,1	30,3	30,5	35,8
		31,4	30,4	30,4	
**CV-B4 n°5**	31,7	31,0	30,9	33,0	36,4
	31,7	31,1	30,9	32,0	35,8
		30,8	31,1	32,8	
**CV-B4 n°6**	29,9	29,8	29,7	30,6	36,3
	29,9	29,8	29,8	31,1	n/a
		29,4	30,0	31,2	

Biological replicates used	11 (day2)<br>12 (day 3)<br>12 (day 7)	11 (day 2)<br>11 (day 3)<br>11 (day 7)	11 (day 2)<br>12 (day 3)<br>12 (day 7)	11 (day 2)<br>12 (day 3)<br>10 (day 7)

Supplementary Table 1. Raw Ct of *Igf2* expression in Figure 6B at day 2, 3 and 7 P.I. Grey, outlined sample or value deleted from analysis; n/a: under the limit of detection. The second table indicate the number of biological replicates used in the analysis of the experiment.

Supplementary Table 2. Putative transcription factor binding sites in the region -68/-22 and -168/-116.

		**Murine *Igf2* P3 (-68 to -22)**	
**Name**	**Motif ID**	**GeneID**	**Score**
Klf6	M08857_2.00	ENSMUSG00000000078	16.87
Klf4	M08857_2.00	ENSMUSG00000003032	16.87
Klf5	M08857_2.00	ENSMUSG00000005148	16.87
Klf7	M08857_2.00	ENSMUSG00000025959	16.87
Klf3	M08857_2.00	ENSMUSG00000029178	16.87
Klf1	M08857_2.00	ENSMUSG00000054191	16.87
Klf2	M08857_2.00	ENSMUSG00000055148	16.87
Klf12	M08857_2.00	ENSMUSG00000072294	16.87
Klf15	M08907_2.00	ENSMUSG00000030087	16.49
Sp1	M09016_2.00	ENSMUSG00000001280	16.03
Sp4	M09016_2.00	ENSMUSG00000025323	16.03
Sp3	M09016_2.00	ENSMUSG00000027109	16.03
Sp6	M09016_2.00	ENSMUSG00000038560	16.03
Sp8	M09016_2.00	ENSMUSG00000048562	16.03
Sp9	M09016_2.00	ENSMUSG00000068859	16.03
Sp5	M09016_2.00	ENSMUSG00000075304	16.03
Patz1	M08864_2.00	ENSMUSG00000020453	15.798
Zfp341	M08892_2.00	ENSMUSG00000059842	15.256
Zfp263	M08272_2.00	ENSMUSG00000022529	14.357
Klf8	M08871_2.00	ENSMUSG00000041649	14.828
Nkx2-1	M08136_2.00	ENSMUSG00000001496	13.307
Nkx2-5	M08136_2.00	ENSMUSG00000015579	13.307
Nkx2-2	M08136_2.00	ENSMUSG00000027434	13.307

Nkx2-6	M08136_2.00	ENSMUSG00000044186	13.307
Nkx2-3	M08136_2.00	ENSMUSG00000044220	13.307
Nkx2-4	M08136_2.00	ENSMUSG00000054160	13.307
Nkx2-9	M08136_2.00	ENSMUSG00000058669	13.307

Murine *Igf2* P3 (-168 to -116)

Name	Motif ID	GeneID	Score
Zfp263	M07844_2.00	ENSMUSG00000022529	16.224
Sp1	M09016_2.00	ENSMUSG00000001280	15.157
Sp4	M09016_2.00	ENSMUSG00000025323	15.157
Sp3	M09016_2.00	ENSMUSG00000027109	15.157
Sp6	M09016_2.00	ENSMUSG00000038560	15.157
Sp8	M09016_2.00	ENSMUSG00000048562	15.157
Sp9	M09016_2.00	ENSMUSG00000068859	15.157
Sp5	M09016_2.00	ENSMUSG00000075304	15.157
Patz1	M08287_2.00	ENSMUSG00000020453	14.129
Zfp263	M08082_2.00	ENSMUSG00000022529	14.251
E2f4	M09034_2.00	ENSMUSG00000014859	14.084
E2f3	M09034_2.00	ENSMUSG00000016477	14.084
E2f2	M09034_2.00	ENSMUSG00000018983	14.084
E2f1	M09034_2.00	ENSMUSG00000027490	14.084
E2f5	M09034_2.00	ENSMUSG00000027552	14.084
E2f6	M09034_2.00	ENSMUSG00000057469	14.084
Sall4	M08866_2.00	ENSMUSG00000027547	13.786
Prdm5	M08986_2.00	ENSMUSG00000029913	13.783
Fev	M09055_2.00	ENSMUSG00000055197	13.617
Etv3	M09055_2.00	ENSMUSG00000003382	13.617
Etv1	M09055_2.00	ENSMUSG00000004151	13.617
Etv2	M09055_2.00	ENSMUSG00000006311	13.617
Elk3	M09055_2.00	ENSMUSG00000008398	13.617
Gabpa	M09055_2.00	ENSMUSG00000008976	13.617

Elk1	M09055_2.00	ENSMUSG00000009406	13.617
Etv5	M09055_2.00	ENSMUSG00000013089	13.617
Fli1	M09055_2.00	ENSMUSG00000016087	13.617
Etv4	M09055_2.00	ENSMUSG00000017724	13.617
Ets2	M09055_2.00	ENSMUSG00000022895	13.617
Elk4	M09055_2.00	ENSMUSG00000026436	13.617
Elf4	M09055_2.00	ENSMUSG00000031103	13.617
Ets1	M09055_2.00	ENSMUSG00000032035	13.617
Elf1	M09055_2.00	ENSMUSG00000036461	13.617
Elf2	M09055_2.00	ENSMUSG00000037174	13.617
Erg	M09055_2.00	ENSMUSG00000040732	13.617
Erf	M09055_2.00	ENSMUSG00000040857	13.617
Zfp383	M07689_2.00	ENSMUSG00000099689	13.452
Maz	M08988_2.00	ENSMUSG00000030678	13.087
Zfp281	M08999_2.00	ENSMUSG00000041483	13.023
